# Evolutionary Origins and Dynamics of Octoploid Strawberry Subgenomes Revealed by Dense Targeted Capture Linkage Maps

**DOI:** 10.1093/gbe/evu261

**Published:** 2014-12-04

**Authors:** Jacob A. Tennessen, Rajanikanth Govindarajulu, Tia-Lynn Ashman, Aaron Liston

**Affiliations:** ^1^Department of Integrative Biology, Oregon State University; ^2^Department of Biological Sciences, University of Pittsburgh; ^3^Department of Botany and Plant Pathology, Oregon State University

**Keywords:** Fragaria, polyploidy, phylogenetics, introgression, transposition, genome assembly

## Abstract

Whole-genome duplications are radical evolutionary events that have driven speciation and adaptation in many taxa. Higher-order polyploids have complex histories often including interspecific hybridization and dynamic genomic changes. This chromosomal reshuffling is poorly understood for most polyploid species, despite their evolutionary and agricultural importance, due to the challenge of distinguishing homologous sequences from each other. Here, we use dense linkage maps generated with targeted sequence capture to improve the diploid strawberry (*Fragaria vesca*) reference genome and to disentangle the subgenomes of the wild octoploid progenitors of cultivated strawberry, *Fragaria virginiana* and *Fragaria chiloensis.* Our novel approach, POLiMAPS (Phylogenetics Of Linkage-Map-Anchored Polyploid Subgenomes), leverages sequence reads to associate informative interhomeolog phylogenetic markers with linkage groups and reference genome positions. In contrast to a widely accepted model, we find that one of the four subgenomes originates with the diploid cytoplasm donor *F. vesca,* one with the diploid *Fragaria iinumae*, and two with an unknown ancestor close to *F. iinumae*. Extensive unidirectional introgression has converted *F. iinumae*-like subgenomes to be more *F. vesca*-like, but never the reverse, due either to homoploid hybridization in the *F. iinumae*-like diploid ancestors or else strong selection spreading *F. vesca*-like sequence among subgenomes through homeologous exchange. In addition, divergence between homeologous chromosomes has been substantially augmented by interchromosomal rearrangements. Our phylogenetic approach reveals novel aspects of the complicated web of genetic exchanges that occur during polyploid evolution and suggests a path forward for unraveling other agriculturally and ecologically important polyploid genomes.

## Introduction

Whole-genome duplication has occurred frequently in the evolution of flowering plants as well as other taxa, which has resulted in genomes composed of multiple homeologous subgenomes (Otto and Whitton 2000; Wood et al. 2009; Jiao et al. 2011). Polyploidy can evolve through interspecific hybridization (allopolyploidy) or within a single lineage (autopolyploidy), and higher-order polyploids (>4*x*) may have a complex history of multiple allo- and/or autopolyploid duplications in addition to homoploid hybridization events that do not change chromosome number (Marcussen et al. 2014). Following duplication, evolutionarily successful polyploids rapidly undergo radical genomic changes including gene loss, gene conversion, transposition, and increasing diploidization (Fontdevila 2005; Madlung et al. 2005; Chen and Ni 2006; Woodhouse 2010; Feldman and Levy 2012). These dynamic changes compensate for the biochemical “genomic shock” of suddenly having multiple, perhaps divergent copies of all genes, while retaining the fitness benefits of a large, diverse, and versatile genome (Chen and Ni 2006; Hollister et al. 2012). Introgression of DNA from one allo-subgenome to another can be due to homoploid hybridization between the diploid ancestors prior to polyploidization (Marcussen et al. 2014), or it can occur postpolyploidization through homeologous exchange that may be initially reciprocal (crossing over) or nonreciprocal (gene conversion) (Kovarik et al. 2004; Wang et al. 2009; Gaeta and Pires 2010; Salmon et al. 2010; Chalhoub et al. 2014). Transposition mediated by transposable elements occurs often in plants and other organisms (Lisch 2013), so polyploid subgenomes will likely show variation in copy number at loci that duplicated either in an ancestor prior to allopolyploidization or else in the polyploid as a direct response to whole-genome duplication (Chen and Ni 2006). Polyploidy plays a central role in speciation and adaptation, but the evolutionary relationships and genomic interactions among subgenomes are rarely well understood outside of a few heavily studied agriculturally important species (e.g., Xiong and Pires 2011; Feldman and Levy 2012; Page et al. 2013; Marcussen et al. 2014), due to the technical challenge of separating homeologous sequence. Phylogenetic relationships of homeologous chromosomes have been estimated from cytological segregation patterns and FISH (fluorescence in situ hybridization) karyotyping (e.g., Bringhurst 1990; Maluszynska and Hasterok 2005; Lipman et al. 2013), or from individual gene sequences (e.g., Senchina et al. 2003; Shimizu-Inatsugi et al. 2009; Rousseau-Gueutin et al. 2009; Cenci et al. 2012; DiMeglio et al. 2014). However, a full picture of the duplication and subsequent evolution of genomes requires genome-scale sequence data sets with representative markers spanning entire chromosomes.

The strawberries (*Fragaria*) present an excellent system for studying the evolution of polyploidy (Liston et al. 2014). Within a short time (1–4 Ma), a diploid (2*n* = 2*x* = 14 chromosomes) ancestral *Fragaria* has diversified into 20 species, nearly half of which are polyploid (Njuguna et al. 2013). Two wild octoploids, *Fragaria virginiana* and *Fragaria chiloensis*, are sister species and the progenitors of the cultivated octoploid strawberry *F.* × *ananassa* (Njuguna et al. 2013)*.* Phylogenetics based on whole chloroplast genome sequencing places these two species and the octoploid/decaploid *Fragaria iturupensis* as sister to the North American diploid *Fragaria vesca* ssp. *bracteata,* suggesting that the latter is the cytoplasmic donor to the higher-order polyploids (Njuguna et al. 2013). Phylogenetic analysis of nuclear genes indicates that the octoploids have an allopolyploid history with ancestry in both the *F. vesca* clade and a clade containing the Japanese diploid *Fragaria iinumae* (Rousseau-Gueutin et al. 2009). The octoploid subgenomes are highly diploidized and inheritance is thought to be primarily disomic (Byrne and Jelenkovic 1976; Bringhurst 1990; Ashley et al. 2003; Rousseau-Gueutin et al. 2008), although there is some evidence for a small amount of polysomic inheritance (Lerceteau-Köhler et al. 2003). It remains unclear whether subgenomes largely act as independent evolutionary units or whether polysomic recombination over many generations blurs this distinction. Multivalent pairings observed in interspecific hybrids have been used to infer relationships among subgenomes. The most widely accepted cytological octoploid genome formula is AAA′A′BBB′B′ based on segregation patterns (Bringhurst 1990; Sargent et al. 2012) and phylogenetic analysis of individual genes (Rousseau-Gueutin et al. 2009; DiMeglio et al. 2014). However, numerous cytological studies over the past century have suggested various contradictory models (Ichijima 1926; Yarnell 1931; East 1934; Federova 1946; Senanayake and Bringhurst 1967; Nathewet et al. 2010). Although genome-scale methods have been applied to octoploid *Fragaria* (e.g., Isobe et al. 2013; Hirakawa et al. 2014; van Dijk et al. 2014), these studies did not isolate phylogenetic markers differentiating the subgenomes. Thus, the relationships among homeologous chromosomes remain unclear. The size of the octoploid *Fragaria* genome is approximately 80% of a strict quadrupling of the diploid genomes (Hirakawa et al. 2014), suggesting that substantial gene loss has occurred postpolyploidization. Genetic studies of polyploid *Fragaria* will be facilitated by clear demarcations of the subgenomes, essential for both basic and applied research in this evolutionarily and agriculturally important genus (Liston et al. 2014).

**Fig. 1.—:**
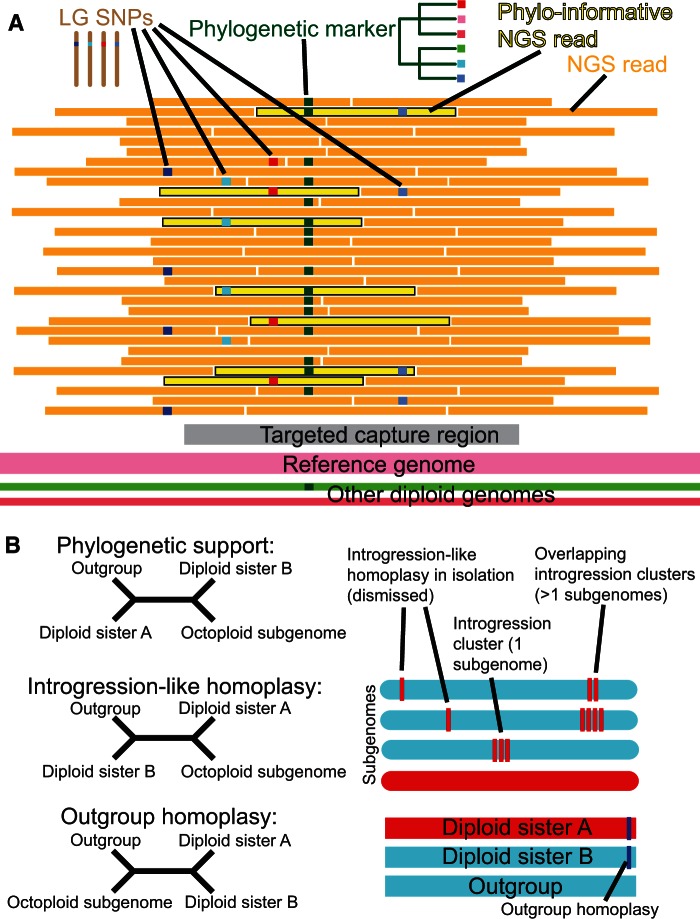
Schematic of our POLiMAPS approach. (*A*) Next-generation sequence reads from octoploids obtained through targeted capture are aligned to our reference genome assembly. LG SNPs occur at approximately one-eighth frequency in a single parent and show Mendelian segregation in the offspring. If an LG SNP shares a read with a marker that occurs on multiple linkage groups and/or diploid taxa, this marker can be used in phylogenetics. A majority of reads do not harbor both an LG SNP and a phylogenetic marker, and thus many linkage groups (e.g., dark blue) have missing data for many phylogenetic markers. (*B*) Identification of introgression. Phylogenetic markers can be classified as providing phylogenetic support, showing introgression-like homoplasy, or showing outgroup homoplasy. Multiple adjacent markers showing introgression-like homoplasy, interspersed with few or no phylogenetically supportive markers, are considered to be caused by true introgression.

Here, we leverage several key resources and strategies, including an improved reference genome, dense linkage maps from crosses of highly heterozygous parents, and high-throughput next-generation sequence data, to dissect the subgenomes of octoploid *Fragaria*. We present a novel approach, POLiMAPS (Phylogenetics Of Linkage-Map-Anchored Polyploid Subgenomes; fig. 1), which allows resolution of previously intractable basic questions about polyploid genome organization and is directly applicable to many other important taxa. Our results revise and enhance the accepted model of evolutionary relationships among subgenomes, and highlight the complex history of genetic exchanges that can underlie polyploid genomes.

## Materials and Methods

### New Fvb Genome

We revised the assembly of the *F. vesca* reference genome (v. 1.1, here designated FvH4; Shulaev et al. 2011) based on linkage map data from three parental plants of *F. vesca* ssp. *bracteata* (supplementary table S1, Supplementary Material online). Our justification for this new assembly is 2-fold. First, the high density of segregating polymorphisms in our linkage maps relative to the *F. vesca* ssp. *vesca* × *Fragaria bucharica* linkage map used in the original genome assembly (Sargent et al. 2011) provides us with greater accuracy in ordering and orienting scaffolds. Second, for any true biological differences in genome structure between *F. vesca* ssp. *bracteata* and either *F. vesca* ssp. *vesca* or *F. bucharica*, the *F. vesca* ssp. *bracteata* arrangement is preferred because this subspecies is the cytoplasmic donor to the octoploid clade (Njuguna et al. 2013). We previously described linkage maps from an outbred cross between two individuals from a single population in Oregon (Tennessen et al. 2013), referred to here as Fvb-m and Fvb-p (“maternal” female ovule donor, “paternal” hermaphrodite pollen donor). Here, we additionally generated a linkage map from F1 offspring derived from selfing a hermaphroditic *F. vesca* ssp. *bracteata* plant collected from Lincoln National Forest, Cloudcroft, NM (32.968 N, −105.750 W) (supplementary table S1, Supplementary Material online) (hereafter Fvb-s for “selfed”—both ovule and pollen donor). Following our previously described methodology (Tennessen et al. 2013), we extracted DNA from 43 F1 offspring and target captured sequences using our published probes (Tennessen et al. 2013). Our probes were originally designed as sets of three partially overlapping 100-bp oligonucleotides surrounding 6,575 central targeted polymorphisms. The targeted polymorphisms were not necessarily segregating in any of the plants examined in this study, but we expected that the probes would often capture other single nucleotide polymorphisms (SNPs). Samples were sequenced in multiplex on the Illumina HiSeq 2000 at Oregon State University. We retained 41 offspring with high sequencing coverage for analysis. Sequencing data in FASTQ format are uploaded to the NCBI SRA (Bioproject Accession PRJNA263688). As in Tennessen et al. (2013), we used BWA (Li and Durbin 2009) to map our reads to a supplemented reference genome that included scaffolds left out of FvH4 (v. 1.1) and converted genotypes to vcf format using SAMtools (Li et al. 2009). We retained genotypes as valid only if per-individual depth was at least 20, the Phred-scaled likelihood was 0, and Phred-scaled likelihood for all other potential genotypes was ≥40 (likelihood of other genotype ≤10^−^^4^); otherwise, genotypes were considered missing. We retained putative polymorphisms for linkage mapping if they showed missing genotypes in fewer than eight offspring, did not show greater than 85% of offspring with the same genotype, and had informative parental genotypes consistent with segregation in the offspring.

We used OneMap (Margarido et al. 2007) to generate a linkage map for Fvb-s. Initially, we only used polymorphic sites with no missing genotypes to create the framework linkage map; sites with missing data were subsequently added manually if possible. We used a logarithm of odds (LOD) of 5 to assign segregating polymorphisms to linkage groups, and polymorphisms were assigned to the most likely position within the linkage group based on their LOD score. Genotyping and mapping errors were identified and corrected manually as described in Tennessen et al. (2013).

We compared our Fvb-s map with our previous Fvb-m and Fvb-p maps, and tested whether the position of any polymorphism was inconsistent among maps. We then used these maps to generate a new genome assembly that placed all polymorphisms in linear order based on their linkage map positions. To do so, we assumed that scaffolds were assembled correctly, and therefore we rearranged the order of whole scaffolds rather than breaking scaffolds into sections, unless we had multiple polymorphisms on the same scaffold mapping to different locations. We defaulted to the scaffold order and orientation in FvH4 unless we had conflicting evidence from our linkage maps. We used the *Prunus persica* (peach) genome (International Peach Genome Initiative et al. 2013) to guide two particular types of decision: The specific locations of scaffold splits and the placement of interchromosome translocations that could not be placed unambiguously based on map position. Thus, we only used the *Prunus* genome as a guide when we already needed to alter the FvH4 assembly based on our linkage maps, and never used the *Prunus* genome alone to override the FvH4 assembly. In order to assess whether FvH4 or Fvb more accurately reflects gene order, we compared synteny of both assemblies with that of the *Prunus* genome. We first used BLAT (Kent 2002) to identify putative orthologs. We then examined the order of *Prunus* orthologs in the two *Fragaria* genomes and vice versa. For each assembly, we counted stretches of continuously syntenous genes (pertaining to the same chromosome, even if their order is rearranged).

### Octoploid Maps

In order to reconstruct a detailed evolutionary history of octoploid strawberry subgenomes, we examined two linkage maps from crosses that have previously been described and initially mapped with simple sequence repeat (SSR) markers (supplementary tables S1 and S2, Supplementary Material online): One for *F. virginiana* ssp. *virginiana* (collected from Pennsylvania; Spigler et al. 2008, 2010, 2011) and one for *F. chiloensis* (collected from Oregon; Goldberg et al. 2010). Both crosses consist of the F1 offspring of two unrelated outbred parents (a female ovule donor and a hermaphrodite pollen donor in each cross). We employed targeted capture sequencing which increased the density of segregating polymorphisms by approximately an order of magnitude, provided each linkage group marker (LG SNP) with a clear expected position in the reference genome, and allowed us to identify phylogenetic markers corresponding to specific linkage groups. DNA was extracted from 100 mg fresh young leaf tissue from F1 offspring (73 for *F. virginiana* ssp. *virginiana* and 46 for *F. chiloensis*) using a modified CTAB (cetyltrimethylammonium bromide) procedure (Doyle JJ and Doyle JL 1987). Targeted capture and sequencing were performed as described above and in Tennessen et al. (2013) (Bioproject Accession PRJNA263688). We analyzed 67 *F. virginiana* F1 offspring and 42 *F. chiloensis* F1 offspring with high sequencing coverage (supplementary table S1, Supplementary Material online). In addition, we sequenced the complete genomes of the maternal parent in both octoploid crosses, following our previous methodology for low coverage whole-genome sequencing of *Fragaria* (Tennessen et al. 2013).

For both octoploid crosses, we mapped reads to the Fvb genome assembly using BWA. Because SAMtools cannot call variants in polyploids, we generated a pileup format file for each cross and used a custom Perl script to call polymorphisms (Perl script available at https://github.com/listonlab/POLiMAPS). Our goal was to identify variants occurring with approximately one-eighth frequency (i.e., heterozygous at a single homeolog), which segregated in a Mendelian fashion (e.g., if heterozygous in one parent then heterozygous in approximately half of the progeny). Fixed differences between homeologs can be distinguished from true SNPs because they will not show Mendelian segregation. We coded genotypes as heterozygous if the rare variant occurred at least twice and at ≥2.5% frequency. We coded genotypes as homozygous if there was no polymorphism or if the rare variant occurred a single time and at less than 1.25% frequency, which we considered to be a potential sequencing error. Ambiguous genotypes that did not meet these criteria or which had less than 32× coverage were coded as missing. To filter for Mendelian segregation, we only retained sites in which the two genotypes were each observed in at least eight progeny. We retained markers for linkage mapping (LG SNPs) only if they had missing data for no more than a single progeny. We converted remaining genotypes to OneMap format and used OneMap to generate linkage maps as with Fvb-s. Due to the complexity of octoploid linkage groups, we did not attempt to manually add LG SNPs with larger numbers of missing genotypes, as we did with the diploid Fvb-s. For both octoploid species, we used a LOD of 5 to assign LG SNPs to linkage groups, and LG SNPs were assigned to the most likely position within the linkage group based on their LOD score. Some linkage groups were then manually joined based on visual inspection of segregation patterns, Fvb position and phylogenetic position. “Minor” linkage groups with fewer than five LG SNPs are reported but excluded from subsequent analysis. For all linkage groups, we assigned a haploid chromosome number (1–7) corresponding to the Fvb pseudochromosome with the largest number of LG SNPs in the linkage group. Each “major” linkage group of ≥5 LG SNPs was then named for the species, haploid chromosome number (Roman numeral matching previous map designations; Goldberg et al. 2010; Spigler et al. 2010; supplementary table S2, Supplementary Material online), subgenome (determined phylogenetically; see below), and parent (p for paternal or m for maternal); for example, “Fvirg-IV-Av-p” is a linkage group from *F. virginiana,* with most of its LG SNPs coming from Fvb4 (and previously designated IV by Spigler et al. 2010), with phylogenetic subgenome designation Av, and with paternally segregating LG SNPs. For most analyses, we ignored whether SNPs fell within the targeted region or in additional nontargeted captured sequence. Adjacent SNPs less than 1 kb apart (approximately the width of adjacent high-coverage sequence captured by a set of overlapping probes) were considered to be distinct LG SNPs but in the same “region,” whether or not that region was targeted with our probes. For some analyses (described below), we restricted the data to only targeted regions.

### Phylogenetic Analysis

For all LG SNPs in the octoploid linkage groups, we identified all original octoploid Illumina reads that mapped to Fvb and contained the minor variant (fig. 1*A*). We used these reads to infer the sequence of the subgenome in the vicinity of the LG SNP (Perl script available at https://github.com/listonlab/POLiMAPS). In cases where a linkage group had an LG SNP less than a read length away from a variant differentiating other linkage groups and/or diploid taxa, we used this adjacent variant as a phylogenetic marker. We did not use the LG SNPs themselves in phylogenetic analysis because they are variable within the linkage group and they were chosen such that the minor variant only occurred in a single subgenome, so we did not expect any meaningful phylogenetic signal from the LG SNPs.

We sequenced whole genomes of *F. iinumae*, *Fragaria mandshurica*, and *Fragaria viridis* (supplementary table S1, Supplementary Material online) for use in phylogenetic analysis, following our previous methodology for low coverage whole-genome sequencing of *Fragaria* (Tennessen et al. 2013) (Bioproject Accession PRJNA263688). Using BWA, we mapped reads from these three species, as well as our original Fvb-s, Fvb-m, and Fvb-p reads, to Fvb and generated a vcf file with SAMtools. In addition, we included the published contigs of *Fragaria nipponica* (http://www.ncbi.nlm.nih.gov/assembly/GCA_000512025) and *F. bucharica* (http://www.ncbi.nlm.nih.gov/assembly/GCA_000511995) and used BLAT with default parameters to match contigs to Fvb (USDA accession CFRA522 is *F. bucharica* but was identified as *Fragaria nubicola* in Hirakawa et al. 2014). Because the octoploid subgenomes originated from a *F. vesca*-like ancestor and a *F. iinumae*-like ancestor, we refer to these two species along with the octoploid subgenomes as “ingroup” taxa, whereas all diploid species other than *F. vesca* and *F. iinumae* are “outgroup” species, even though the ingroup taxa do not form a monophyletic group with each other. As an outgroup to the entire genus, we chose the transcriptome of *Rubus coreanus* (http://www.ncbi.nlm.nih.gov/sra/SRX347804; Hyun et al. 2014). Because *Rubus* is too distant from *Fragaria* to reliably map reads with BWA, we used BLAT with default parameters to match reads to Fvb. We attempted to use *Potentilla micrantha* as an additional outgroup based on nuclear reads from the sequencing of its chloroplast genome (Ferrarini et al. 2013) but coverage was too low to be useful. Using RAxML (Stamatakis 2006) with -N autoMRE and -m GTRCAT, we estimated separate phylogenies for each of the seven haploid *Fragaria* chromosomes from each of the four parents of the octoploid maps and the three parents of the diploid maps (matrices and trees available: http://purl.org/phylo/treebase/phylows/study/TB2:S15849).

### Introgression

We tested for evidence of introgression between *F. vesca*-like and *F. iinumae*-like subgenomes, as can occur through homoploid hybridization before polyploidization or homeologous exchange after polyploidization. Because introgression will manifest as inconsistencies in the phylogenetic signal, we first identified sites showing homoplasy. For a site with a known base for at least one octoploid subgenome, *F. vesca*, *F. iinumae*, and at least one outgroup species, there are three possible phylogenetic patterns (fig. 1*B*). First, the site would be supportive of the phylogeny if the subgenome shares a base with its sister ingroup species (either *F. vesca* or *F. iinumae*), whereas the other two species share a different base. Second, the site would show “introgression-like homoplasy” if the subgenome shares a base with the wrong ingroup species, whereas its sister species shares a different base with the outgroup, consistent with introgression of DNA between the two ingroup lineages. Third, the site would show “outgroup homoplasy” if the subgenome shares a base with the outgroup, whereas *F. vesca* and *F. iinumae* share a different base. In the absence of true introgression, homoplasy is only due to factors such as independent mutations, incomplete lineage sorting, and sequencing errors, and thus introgression-like homoplasy should occur at the same prevalence as outgroup homoplasy. Therefore, we tested for an excess of introgression-like homoplasy in order to detect introgression (similar to the ABBA/BABA test; Green et al. 2010).

For probes with introgression-like homoplasy, we took two additional steps to distinguish between true introgression and spurious homoplasy due to other evolutionary processes such as repeated independent mutations. First, probes with both supportive and introgression-like homoplasy sites for the same parental sample were classified as “support/homoplasy” probes and were considered to not show true introgression. Second, we identified clusters of at least two adjacent introgression-like homoplasy probes in the Fvb genome, such that the ratio of introgression homoplasy probes to supportive probes in the region was at least 4. Only these clusters were considered to be true introgression events. In order to assess whether introgression occurred before or after polyploidization, we tested whether introgressed chromosomes showed a closer phylogenetic relationship with their homeologous chromosomes or with the sister ingroup diploid species.

As an independent test of introgression, we examined the relative depth of *F. vesca*-like and *F. iinumae*-like alleles across the low-coverage genomes of the maternal parents of both octoploid crosses. We identified SNPs distinguishing *F. vesca* and *F. iinumae* that were also polymorphic in the octoploids, with a combined depth across the two maternal parents between 45× and 200×. We calculated the depth ratio for both variants, excluding sites with a ratio greater than 8 or less than 1/8 as likely errors, and tested whether this ratio differed between introgression clusters and the rest of the genome.

To look for correlations between overall depth and introgression, we measured depth at the central targeted site of each targeted region in the Fvb-s parent and the octoploid parents. We normalized depth for each individual by dividing by the sum of depths at all central targeted sites, and for each targeted region we calculated the mean normalized depth across octoploids. We then calculated the ratio of normalized depth in octoploids versus Fvb-s, and we tested whether this ratio differs among regions with or without introgression.

### Interchromosomal Rearrangements

In order to identify interchromosome rearrangements, we first incorporated data from a recent linkage map of *F.* × *ananassa* (Isobe et al. 2013) along with our octoploid linkage maps. We used the primer sequences for each SSR marker (Isobe et al. 2013) and we performed in silico polymerase chain reaction (PCR) with BLAT (Kent 2002) on our Fvb assembly. We retained markers for which both forward and reverse primers mapped within 2 kb of each other. To assess translocations among subgenomes and species, we assigned all linkage groups in *F.* × *ananassa*, *F. virginiana* ssp. *virginiana*, and *F. chiloensis* to a haploid chromosome that matched the majority of its markers (SSR or SNPs), and then we identified markers with an Fvb position that did not match this primary chromosome. We tested whether any translocations matched FvH4 but not Fvb, which could indicate errors in our Fvb assembly. We tested whether rearrangements were significantly clustered in their reference genome position by generating 10,000 simulated data sets in which LG SNPs showing rearrangements were randomly distributed among all targeted regions with LG SNPs. As with introgression (above), we tested whether the ratio of octoploid:diploid normalized depth differed between targeted regions with or without interchromosomal rearrangements.

## Results

### *New Fragaria* Reference Genome

In the targeted capture sequence data from the 41 Fvb-s progeny, 3.244 Mb had a mean per-individual depth ≥20× . Within these high-coverage sites, 1,825 polymorphic sites passed our quality thresholds and were used in linkage mapping. Our initial Fvb-s linkage map consisted of eight linkage groups corresponding to the seven *Fragaria* chromosomes with two linkage groups for different sections of FvH4 pseudochromosome 4, which were manually joined for a final Fvb-s linkage map of seven linkage groups spanning 326 cM.

**Fig. 2.—:**
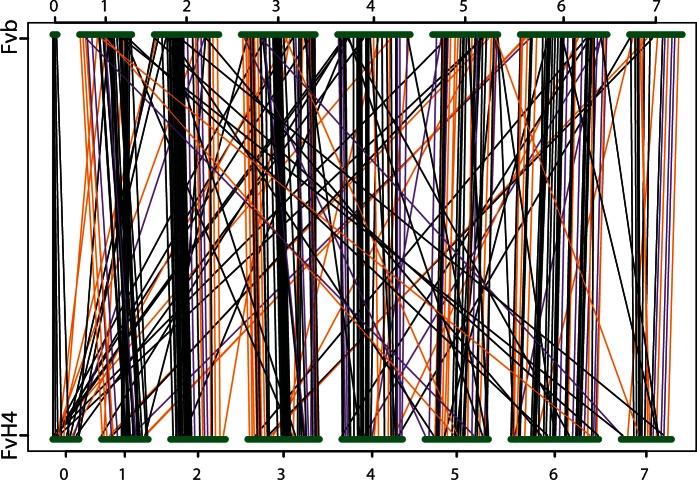
Mapping of scaffolds from FvH4 reference genome assembly to Fvb assembly. The path of every scaffold or scaffold segment is represented by a line (orange, inverted in diploid linkage maps; purple, not inverted in diploid linkage maps; black, no information about scaffold orientation in diploid linkage maps, retained default noninverted orientation).

**Fig. 3.—:**
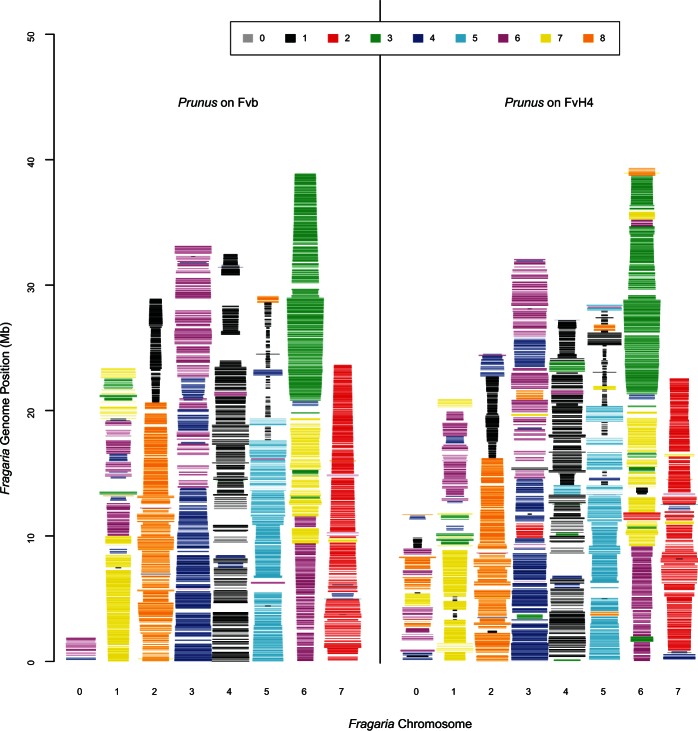
*Prunus* orthologs mapped onto *Fragaria* assemblies. Each horizontal line represents an orthologous gene, colored according to its *Prunus* chromosome, and with a width corresponding to its position on this *Prunus* chromosome (wider lines are close to the start of their respective *Prunus* chromosome). Its *x* axis position indicates the *Fragaria* pseudochromosome onto which it is assembled. Its *y* axis position indicates its position on this *Fragaria* pseudochromosome. Thus, regions showing lines of the same color and similar width indicate high synteny between the two genera. Overall, Fvb shows higher synteny than FvH4.

We found no discrepancies among the Fvb-m, Fvb-p, and Fvb-s linkage maps. In contrast, between FvH4 and our *F. vesca* ssp. *bracteata* maps, we observed 44 interchromosome translocations, 40 intrachromosome translocations, 39 inversions, and 18 placements of unmapped FvH4_0 scaffolds (87 of these 141 rearrangements are the same as those previously described, Tennessen et al. 2013). We made 39 splits among the 246 FvH4 scaffolds and added two missing scaffolds (scA and scB as described in Tennessen et al. 2013) for a total of 287 new scaffolds, which we rearranged to match the *F. vesca* ssp. *bracteata* maps (fig. 2; supplementary table S3, Supplementary Material online). Our new assembly, here designated Fvb, contains 208.9 Mb of scaffold sequence, with 207.0 Mb assembled into seven pseudochromosomes (Fvb1–Fvb7) and 1.9 Mb remaining unassembled on a false chromosome Fvb0. With 10-kb gaps separating all scaffolds, the complete size of Fvb is 211.7 Mb (Available on Figshare: http://dx.doi.org/10.6084/m9.figshare.1259206). We observed 164 distinct stretches of contiguous *Prunus* orthologs in FvH4, but only 116 such stretches in Fvb (fig. 3).

### Octoploid Maps

**Fig. 4.—:**
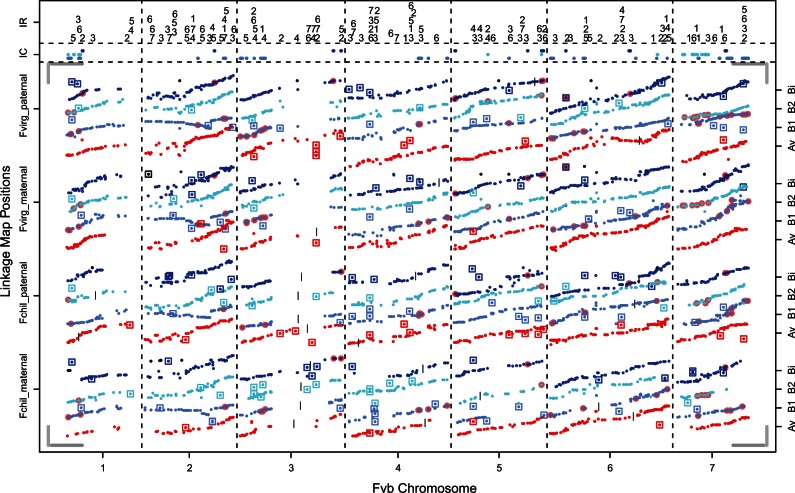
Linkage maps for *F. chiloensis* and *F. virginiana* ssp. *virginiana*, aligned to the Fvb reference genome. Segregating LG SNPs are represented by small solid circles. For all LG SNPs, the *y* axis position corresponds to linkage map position (cM) and the *x* axis position corresponds to Fvb genome position (Mb). All seven Fvb chromosomes are represented on the *x* axis, with scale indicated by dark gray horizontal bars in all four corners (=10 Mb). All four parents are represented on the *y* axis, with scale indicated by light gray vertical bars in all four corners (=100 cM). Linkage groups are colored based on their phylogenetic position (red, Av; cornflower, B1; cyan, B2; blue, Bi). LG SNPs in black correspond to minor linkage groups (<5 LG SNPs) that could not be placed in the phylogeny. Short vertical black bars indicate junctures where smaller linkage groups were manually joined based on visual inspection of genotypes, phylogenetic position, and Fvb position. Introgression clusters are shown as light red circles behind LG SNPs, and their positions are again shown along the top of the figure (IC), colored according to subgenome. Interchromosome rearrangements are shown as squares around LG SNPs, and their positions are again shown along the top of the figure (IR), with numbers indicating the haploid chromosome of the linkage group.

In the targeted capture reads from the two *F. virginiana* ssp. *virginiana* parents and their 67 progeny, 1,506 kb had a mean per-individual depth ≥32×, whereas 553 kb had a mean per-individual depth ≥80×. A total of 3,875 LG SNPs passed our quality thresholds and were used in linkage mapping, including 1,899 maternal LG SNPs and 1,976 paternal LG SNPs. The additional 594 LG SNPs polymorphic in both parents were not placed in the linkage maps but were used to identify pairs of corresponding linkage groups between the parents. Using a LOD threshold of 5 initially yielded 27 maternal linkage groups and 29 paternal linkage groups. We split all linkage groups anywhere there was a gap greater than 33 cM, resulting in a 34 maternal linkage groups and 35 paternal linkage groups. Some of these linkage groups were then manually joined based on visual inspection of segregation patterns, position of these groups of LG SNPs in the Fvb assembly (e.g., if they corresponded to the same Fvb pseudochromosome), and phylogenetic position (see below). Our final count was thus 28 major linkage groups ( ≥ 33 LG SNPs each) for both parents, representing the 28 haploid chromosomes, plus 4 maternal and 5 paternal minor linkage groups ( ≤ 3 LG SNPs each) (fig. 4).

In the targeted capture reads from the two *F. chiloensis* parents and their 42 progeny, 1,632 kb had a mean per-individual depth ≥32× , whereas 705 kb had a mean per-individual depth ≥80× . A total of 2,542 LG SNPs passed our quality thresholds and were used in linkage mapping, including 1,100 maternal LG SNPs and 1,442 paternal LG SNPs. As with *F. virginiana* ssp. *virginiana*, an additional 780 LG SNPs polymorphic in both parents were used to pair corresponding linkage groups. Using a LOD threshold of 5 initially yielded 39 maternal linkage groups and 36 paternal linkage groups. Splitting at gaps greater than 33 cM resulted in 39 maternal linkage groups and 40 paternal linkage groups. As with *F. virginiana* ssp. *virginiana*, some of these linkage groups were then manually joined, resulting in 28 major linkage groups ( ≥17 LG SNPs each) for both parents, representing the 28 haploid chromosomes, plus 2 maternal and 2 paternal minor linkage groups ( ≤ 4 LG SNPs each) (fig. 4).

### Phylogenetic Analysis

**Fig. 5.—:**
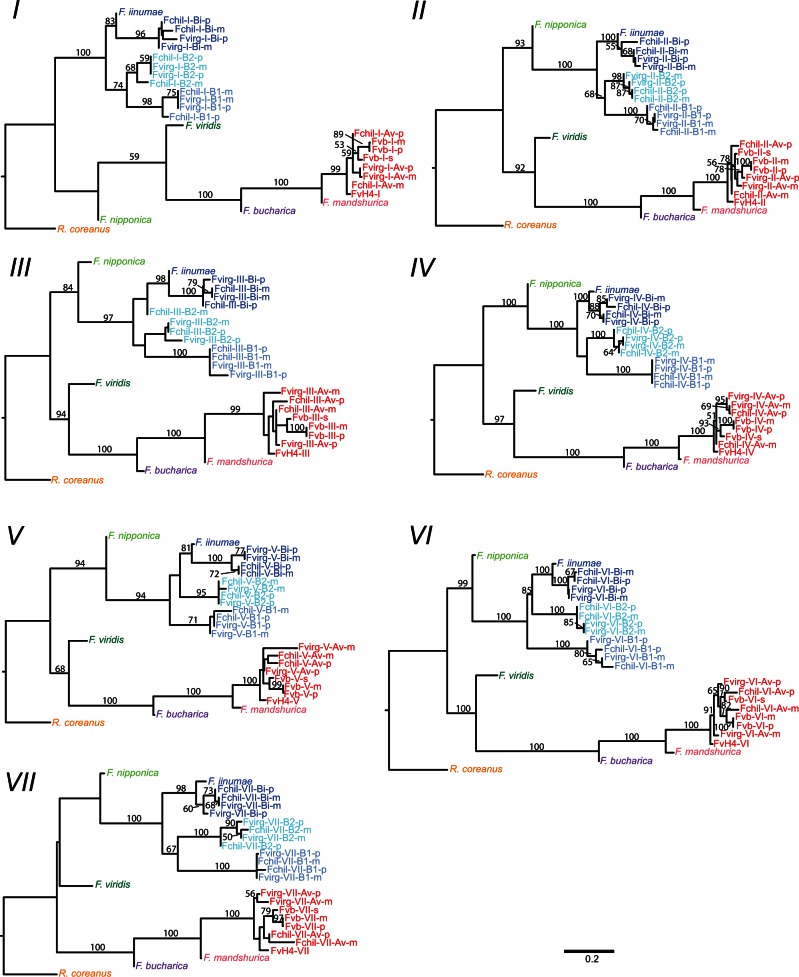
Phylogenies of all seven haploid *Fragaria* chromosomes. Octoploid subgenomes are named for the species (Fchil or Fvirg), haploid chromosome number (Roman numeral), subgenome (Av, B1, B2, or Bi), and parent (p or m), and are colored as in figure 4 with *F. iinumae* (blue) and *F. vesca* (FvH4, Fvb-m, Fvb-p, and Fvb-s; red) similarly colored. Other diploid taxa have distinct colors. Numbers on branches represent bootstrap values; bootstraps less than 50 or on branches with length less than 0.01 substitutions/informative site are not shown.

We obtained 540–1,517 markers per haploid chromosome for phylogenetic analysis, 317–902 of which were phylogenetically informative with respect to the relationship between an octoploid subgenome, *F. vesca*, *F. iinumae*, and one of the diploid outgroup species (table 1 and supplementary table S1, Supplementary Material online). These markers represented 160–421 regions of 1 kb in Fvb (table 1). For any given site, we could only include a linkage group if it had an LG SNP less than read length away (fig. 1*A*), so at most sites the majority of taxa had missing data (64% of total data missing). Nevertheless, phylogenetic signal was sufficient to resolve trees with high bootstrap support (usually greater than 90%) for the major clades at all seven chromosomes (fig. 5; matrices and trees available: http://purl.org/phylo/treebase/phylows/study/TB2:S15849). In all seven trees, one of the four octoploid subgenomes groups with *F. vesca*, which we designated subgenome Av. Chromosomes from the other three subgenomes consistently form a clade with *F. iinumae.* The subgenome sister to *F. iinumae* we designated subgenome Bi, and the two basal to the *F. iinumae*/Bi clade we designated subgenomes B1 and B2, with B1 being the subgenome showing greatest divergence from *F. iinumae*. Because this divergence criterion is weak relative to the topology-based criteria, this distinction may not be evolutionarily meaningful across haploid chromosomes of subgenomes B1 versus B2.

**Table 1:** Regions of 1-kb Showing Phylogenetic Support and Homoplasy

Phylogenetic Signal	Subgenome	I LGs	II LGs	III LGs	IV LGs	V LGs	VI LGs	VII LGs	Total
Supportive^a^									
	Av	54	120	55	97	118	198	90	732
	Bi	44	117	78	93	91	175	102	700
	B1	60	104	43	67	84	120	72	550
	B2	61	104	46	91	86	113	59	560
Clustered introgression-like (clusters)^b^									
	Av	0 (0)	0 (0)	0 (0)	0 (0)	0 (0)	0 (0)	0 (0)	0 (0)
	Bi	2 (1)	4 (2)	4 (2)	0 (0)	11 (4)	2 (1)	5 (1)	28 (11)
	B1	10 (2)	7 (2)	16 (5)	15 (3)	2 (1)	19 (8)	25 (5)	94 (26)
	B2	6 (3)	0 (0)	0 (0)	0 (0)	4 (2)	3 (1)	17 (5)	30 (11)
Isolated introgression-like^c^									
	Av	2	4	3	6	4	8	7	34
	Bi	6	4	7	6	11	8	3	45
	B1	4	7	5	6	3	17	12	54
	B2	5	5	7	9	5	10	9	50
Outgroup homoplasy^d^									
	Av	2	6	3	6	6	10	18	51
	Bi	1	2	4	3	3	10	7	30
	B1	2	7	5	5	3	14	16	52
	B2	8	6	5	2	10	19	13	63
Support/homoplasy^e^									
	Av	1	5	3	6	5	7	4	31
	Bi	1	6	1	3	4	8	8	31
	B1	3	9	3	8	6	16	10	55
	B2	2	10	6	5	10	11	13	57
Total informative regions^f^	All	160	298	185	230	255	421	264	1,813
Total informative markers^g^	All	317	645	367	483	568	902	536	3,818
Total markers^h^	All	540	1,152	627	784	905	1,517	956	6,481
Total missing^i^	All	64.5	64.5	64.5	63.2	64.1	63.9	64.6	64.2

^a^Regions which support the phylogenetic position of a given subgenome.

^b^Regions showing homoplasy such that a subgenome’s relationships to *Fragaria iinumae* and *Fragaria vesca* are switched, which occur in clusters near other such regions. We conclude that these are true introgression events.

^c^Regions showing homoplasy such that a subgenome’s relationships to *F. iinumae* and *F. vesca* are switched, which do not occur in clusters. These may be additional introgression events but we do not analyze them as such.

^d^Regions showing homoplasy such that a subgenome matches an outgroup taxon and not *F. iinumae* and *F. vesca.*

^e^Regions with sites that both support the position of a parental sample and show homoplasy for the same parental sample.

^f^Regions containing at least one phylogenetic marker that is informative with respect to the relationship between an octoploid subgenome, *F. vesca*, *F. iinumae*, and a third outgroup taxon (informative markers).

^g^Count of informative markers.

^h^Count of total phylogenetic markers.

^i^Percentage of missing data.

### Introgression

**Fig. 6.—:**
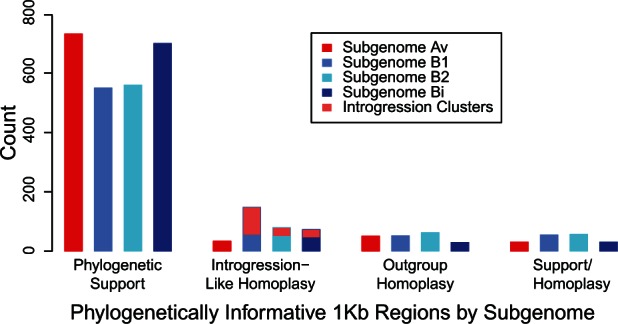
Regions of 1 kb with phylogenetically informative markers. For a phylogeny consisting of an outgroup, *F. vesca*, *F. iinumae*, and a subgenome, there are three possible patterns (fig. 1*B*): Phylogenetic support, introgression-like homoplasy, and outgroup homoplasy. Regions with multiple markers may also show a mix of support and homoplasy. There is an excess of introgression-like homoplasy relative to outgroup homoplasy, but it can be accounted for entirely by the introgression clusters, which occur only on subgenomes B1, B2, and Bi.

**Fig. 7.—:**
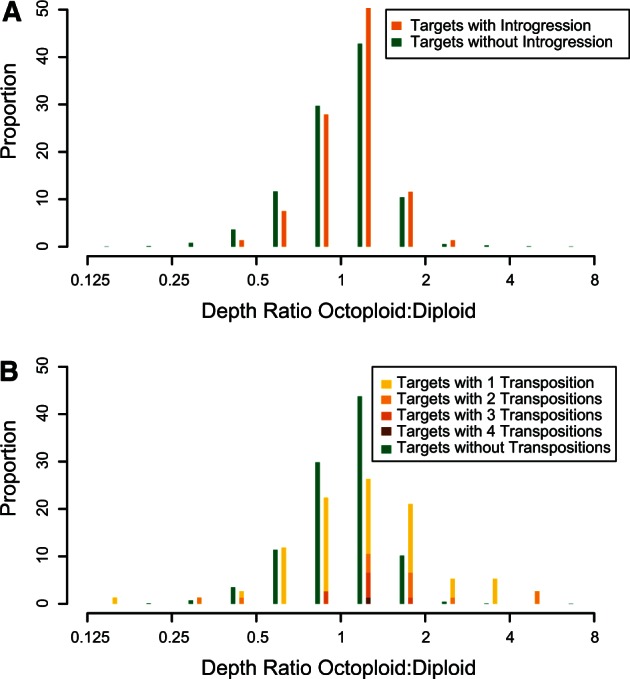
Relative normalized coverage in octoploids relative to diploid, for targeted regions showing dynamic evolutionary changes. Regions are binned on a log scale along the *x* axis with breakpoints at multiples of √2. (*A*) Targeted regions showing introgression do not have significantly different depth than regions not showing introgression. (*B*) Targeted regions showing interchromosome rearrangements have higher and more variable depth in octoploid relative to diploid, indicating that many of these regions undergo “copy-and-paste” transposition, resulting in unequal copy numbers across species.

We observed phylogenetic support at 54–198 regions per haploid chromosome (732 total) for subgenome Av, 44–178 regions (700 total) for subgenome Bi, 43–120 regions (550 total) for subgenome B1, and 46–113 regions (560 total) for subgenome B2 (table 1; fig. 6). We also observed a large number of regions showing homoplasy with respect to the consensus trees, that is, they are inconsistent with the phylogeny: 116 for Av, 134 for Bi, 255 for B1, and 200 for B2 (table 1; fig. 6). To find introgression homoplasy, we considered regions where subgenome Bi, B1, or B2 is closer to *F. vesca* than to *F. iinumae,* or where subgenome Av is closer to *F. iinumae* than to *F. vesca* (fig. 1*B*). Introgression homoplasy was 1.7 times as common as outgroup homoplasy (335 vs. 196; *P* < 0.0001; Fisher’s exact test; table 1). The most common type of homoplasy was when subgenome Bi, B1, or B2 appeared closer to *F. vesca* than to *F. iinumae,* with more than twice as many regions showing this type of homoplasy than would be expected given the outgroup homoplasy (301 vs. 145; *P* < 0.0001; Fisher’s exact test; table 1). Because individual instances of homoplasy could be due to independent mutations or incomplete lineage sorting rather than true introgression, we focused on the 152 regions that formed 48 distinct genomic clusters containing multiple (2–11) regions showing introgression homoplasy (table 2). If these clusters are excluded, there is no longer an excess of introgression homoplasy (fig. 6). These introgression clusters are dispersed throughout the genome, occurring on homeologs of all seven Fvb pseudochromosomes, and encompassing 15.0 Mb (7% of Fvb). Clusters range in size from 2 to 1,579 kb (median = 160 kb; mean = 324 kb). There are only three instances where introgression clusters on different subgenomes overlap, encompassing 604 kb, and introgression regions do not differ from the rest of the genome in relative sequencing coverage (*t*-test, *t* = 2.22, *P* > 0.01; fig. 7*A*).

**Table 2:** Introgression Clusters

Fvb Chromosome^a^	Fvb Sites^b^	Subgenome^c^	Regions^d^	Linkage Maps^e^
1	5065413–5220128	Bi	2	Fvirg-p
1	427431–447178	B2	2	Fchil-p,Fvirg-p
1	2235284–2394395	B2	2	Fvirg-m,Fvirg-p
1	3893160–4468792	B2	2	Fchil-m,Fvirg-m
1	427431–1390854	B1	8	Fchil-m,Fchil-p,Fvirg-m,Fvirg-p
1	3893160–4468792	B1	2	Fvirg-m,Fvirg-p,Fchil-m
2	22607224–22676892	Bi	2	Fvirg-m,Fvirg-p
2	17223334–17302784	B1	2	Fvirg-m,Fvirg-p
2	25578591–26245090	B1	5	Fchil-m,Fchil-p,Fvirg-m,Fvirg-p
2^f^	5110972–5139963	Bi	2	Fvirg-m,Fvirg-p
3	30308416–30469404	Bi	2	Fchil-m,Fvirg-p
3	32852305–32955512	Bi	2	Fchil-m,Fchil-p
3	124040–759546	B1	2	Fchil-p,Fvirg-p
3	2067176–2179483	B1	4	Fvirg-m,Fvirg-p
3	4625730–4646210	B1	2	Fvirg-m,Fvirg-p
3	6728933–6815506	B1	3	Fvirg-m,Fvirg-p,Fchil-m
3	7342503–8226577	B1	5	Fchil-m,Fchil-p,Fvirg-m,Fvirg-p
4	22867544–23893840	B1	10	Fchil-m,Fchil-p,Fvirg-m,Fvirg-p
4	26100382–26165868	B1	2	Fchil-m,Fvirg-m
4	32133852–32406162	B1	3	Fchil-m,Fvirg-m
5	275987–481317	Bi	4	Fvirg-m,Fvirg-p,Fchil-p
5	23910116–23947199	Bi	3	Fchil-m,Fchil-p,Fvirg-m
5	28429618–28438057	Bi	2	Fvirg-m,Fvirg-p
5	28912695–29086611	Bi	2	Fvirg-m,Fvirg-p
5	10990292–10992194	B2	2	Fvirg-m,Fvirg-p
5	28429618–28438057	B2	2	Fchil-m,Fchil-p,Fvirg-p
5	17792748–17936726	B1	2	Fvirg-m,Fvirg-p
6	25670424–26071024	Bi	2	Fchil-p,Fvirg-p
6	34884370–34958872	B2	3	Fchil-p
6	127855–704274	B1	4	Fchil-m,Fchil-p,Fvirg-p
6	1024942–1107448	B1	3	Fvirg-m,Fvirg-p,Fchil-p
6	7767792–7780026	B1	2	Fvirg-m,Fvirg-p
6	10737836–10761718	B1	2	Fvirg-m,Fvirg-p,Fchil-m
6	11163923–11407930	B1	2	Fvirg-m,Fvirg-p,Fchil-p
6	27838353–27895788	B1	2	Fchil-m,Fvirg-m
6	34652543–34656025	B1	2	Fchil-m,Fchil-p
6	38080971–38105006	B1	2	Fchil-m,Fvirg-m
7	16085862–16472060	Bi	5	Fchil-m,Fchil-p,Fvirg-m
7	2247584–2857258	B2	2	Fvirg-m,Fvirg-p
7	5329113–5933718	B2	3	Fvirg-m,Fvirg-p
7	6727176–7011896	B2	4	Fchil-m,Fchil-p,Fvirg-m,Fvirg-p
7	8420008–8481933	B2	2	Fchil-m,Fvirg-m
7	9296554–10605847	B2	6	Fchil-m,Fchil-p,Fvirg-m,Fvirg-p
7	7246522–7567127	B1	3	Fchil-m,Fchil-p,Fvirg-p
7	14121260–14312154	B1	2	Fvirg-m,Fvirg-p
7	16665708–18245006	B1	11	Fchil-m,Fchil-p,Fvirg-m,Fvirg-p
7	21644908–22959952	B1	6	Fchil-m,Fchil-p,Fvirg-p
7	23427780–23594132	B1	3	Fvirg-m,Fvirg-p,Fchil-p

^a^Pseudochromosome in Fvb reference genome.

^b^Physical position in Fvb reference genome.

^c^Subgenome designation of linkage group.

^d^Count of adjacent nonoverlapping approximately 1-kb regions contributing to introgression event.

^e^Parental linkage maps in which introgression event is observed.

^f^Primary Fvb chromosome of linkage group is Fvb6 (interchromosome rearrangement).

**Fig. 8.—:**
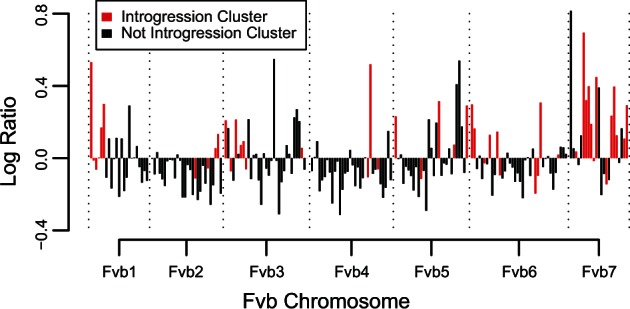
Ratio of *F. vesca*-like read depth to *F. iinumae*-like read depth for the two maternal octoploid parents in 1-Mb bins across the Fvb genome. Bins overlapping introgression clusters as determined from the linkage map data (red) show a much higher ratio. The ratio in all bins is likely skewed toward *F. vesca* because such reads are more likely to map to Fvb.

Strikingly, introgression is unidirectional in that every cluster involves an *F. iinumae*-like subgenome (Bi, B1, or B2) evolving to be more similar to *F. vesca*, with no instances of introgression making subgenome Av more similar to *F. iinumae* (48 vs. 0; compared with the expectation of 36 vs. 12, Fisher’s exact test, *P* < 0.001). More specifically, 54% of introgression clusters involve subgenome B1 acquiring *F. vesca*-like sequence. Because we defined subgenome B1 as having greater divergence from *F. iinumae*, introgression may be confounded with the distinction between B1 and B2. However, the average amount of introgression between B1 and B2 (18.5 clusters, 62 regions, 6.9 Mb) is higher than both Bi (11 clusters, 28 regions, 1.7 Mb) and Av (no introgression). In the low-coverage whole-genome data, the mean ratio of *F. vesca*-like reads to *F. iinumae*-like reads is significantly higher within clusters (1.37) than in the rest of the genome (0.98) (*t*-test, *t* = 21.96, *P* < 10^−^^15^; fig. 8). Across the genome, there are only 48 sites that can inform whether introgressed regions are closer to *F. vesca* or to the Av subgenome, that is, where a *F. iinumae*-like subgenome has a derived allele with respect to outgroup taxa which is shared by either *F. vesca* or the Av subgenome, but not both. Of these, 23 unite the *F. iinumae*-like subgenome with *F. vesca* to the exclusion of the Av subgenomes, and 25 unite the *F. iinumae*-like subgenome with the Av subgenomes to the exclusion of *F. vesca*.

### Interchromosome Rearrangements

We were able to assign 777 SSR markers from *F.* × *ananassa* to Fvb positions. Among the three octoploid species, we observed 139 interchromosomal rearrangements, defined as regions of Fvb in which an LG SNP maps to a linkage group pertaining to a different Fvb pseudochromosome (supplementary table S4, Supplementary Material online; fig. 4). The 139 interchromosomal rearrangements are highly clustered, falling into only 110 1-kb regions of Fvb, 25 of which harbor at least two rearrangements, 9 of which harbor at least three rearrangements, and 1 of which harbors four rearrangements. For 120 rearrangements, we observed direct conflicts among our linkage maps, with LG SNPs from the same 1-kb region mapping to linkage groups pertaining to different Fvb pseudochromosomes. Only 25 rearrangements are supported by at least two LG SNPs, and only 4 by LG SNPs greater than 1 kb apart. Subgenome Av showed fewer rearrangements relative to the other subgenomes, consistent with its closer phylogenetic relationship to Fvb; the exceptions were regions with low mean normalized depth per targeted region in the octoploids versus Fvb-s (putatively duplicated within *F. vesca* ssp. *bracteata*), and nontargeted regions (possibly duplicated throughout *Fragaria*).

To minimize variance in coverage and standardize expected distribution patterns, we restricted further analysis to the 105 rearrangements that occur within 1 kb of one of 76 targeted regions. There are 20 targeted regions with two or more of these rearrangements, and each of the 105 of them occurs on a targeted region with 0.74 other rearrangements on average. However, if 105 rearrangements were randomly distributed among all 2,598 targeted regions with LG SNPs in the octoploids, only two regions with multiple rearrangements are expected (95% CI: 1–3), and the average rearrangement would share a targeted region with only 0.04 others (95% CI: 0.00–0.10), a highly significant difference indicating nonrandom clustering. The ratio of mean normalized depth per targeted region in the octoploids versus Fvb-s was higher and more variable at targeted regions showing rearrangements (mean ratio = 1.34, SD = 0.82) relative to other targeted regions with LG SNPs in the octoploids (mean ratio = 1.04, SD = 0.34; *t*-test, *t* = 3.14, *P* < 0.01), suggesting copy number variation caused by duplication of these regions in some lineages since the common *Fragaria* ancestor (fig. 7*B*).

## Discussion

Using a novel approach, POLiMAPS, we have generated a phylogeny of octoploid homeologous subgenomes. We have successfully recovered and assigned a phylogenetic position to all eight expected linkage groups for every haploid chromosome in two independent octoploid crosses (figs. 4 and 5). Unlike methods based on individual genes (Senchina et al. 2003; Rousseau-Gueutin et al. 2009; Shimizu-Inatsugi et al. 2009; DiMeglio et al. 2014), we employ thousands of genome-wide markers, revealing the evolutionary history of each subgenome as a whole. POLiMAPS enhances mapping-by-sequencing (James et al. 2013) by mapping targeted sequence three ways: To a reference genome, to a linkage group, and to a phylogenetic tree, in order to highlight the evolutionary processes that produce consistencies and discrepancies among these three types of maps. Our results reveal that divergence among homeologs is heavily influenced by dynamic genomic changes affecting many sites at once, including introgression and chromosomal rearrangements.

A fundamental result of this study is that only one subgenome (Av) groups with *F. vesca* in our phylogenies, whereas three subgenomes (Bi, B1, and B2) group with *F. iinumae* (fig. 5). These data allow us to reject the prevailing hypothesis which posits two *F. vesca*-like subgenomes (Rousseau-Gueutin et al. 2009; Sargent et al. 2012). A parsimonious evolutionary history consistent with our results is that a *F. vesca*-like diploid hybridized with a *F. iinumae*-like diploid to form an allotetraploid (AvAvBiBi), which then hybridized with an unknown *F. iinumae*-like autotetraploid (B1B1B2B2) to form the octoploid ancestor of *F. chiloensis* and *F. virginiana*. Our results otherwise support the relationships among *Fragaria* species suggested by previous studies of nuclear (Rousseau-Gueutin et al. 2009) and plastome (Njuguna et al. 2013) sequence. Specifically, we confirm that *F. iinumae* is phylogenetically isolated, that *F. mandshurica* and *F. bucharica* are closely related to *F. vesca*, that the octoploids have a common origin, and that *F. vesca* ssp. *bracteata* has a close, likely conspecific relationship with one of the diploid ancestors of the octoploids (Av). For our analyses we assumed, based on previous work (Rousseau-Gueutin et al. 2009; Njuguna et al. 2013), that other *Fragaria* diploids not sampled are not candidate progenitors of the octoploids. Our results are consistent with the accepted view of disomic inheritance in which homeologous chromosomes are independent and do not cosegregate (Bringhurst 1990; Ashley et al. 2003; Rousseau-Gueutin et al. 2008). Specifically, subgenomes reliably form distinct clades, rather than multiple subgenomes from the same species forming a clade together as would be expected under free recombination among homeologs (Wendel et al. 1995). Although polysomic recombination has not eliminated the phylogenetic signal, suggesting a highly diploidized genome, a small amount of polysomic inheritance may still occur, especially between B1 and B2 (Lerceteau-Köhler et al. 2003).

With POLiMAPS, specific alleles of interest can be placed in a phylogenetic context. For example, prior work has shown that both *F. virginiana* ssp*. virginiana* and *F. chiloensis* have a Mendelian sex locus mapping to different ends of different chromosomes in homeologous group VI (supplementary table S2, Supplementary Material online), suggesting either independent origins or translocation (Goldberg et al. 2010). Our results reveal that the *F. chiloensis* sex locus maps to subgenome Av whereas the *F. virginiana* ssp*. virginiana* sex locus maps to subgenome B2 (supplementary table S2, Supplementary Material online). The “use” of different autosomes for sex chromosomes has been seen among species in the same genera in other taxa (e.g., sticklebacks; Ross et al. 2009) but this is the first study to clearly demonstrate the use of the same chromosomes but from different ancestors. This example demonstrates the power of our phylogenetic model to enhance understanding of trait and chromosomal evolution, which will stimulate future research in strawberry genetics, including for agriculturally important traits, especially with respect to using the *F. vesca* reference genome as a proxy for cultivated strawberry.

Homoplasy consistent with introgression has been reported in many polyploid species, although it can be challenging to conclusively identify and interpret (Gaeta and Pires 2010; Wijnker et al. 2013; Chalhoub et al. 2014; Marcussen et al. 2014; Qi et al. 2014). Detection methods based on sequencing alone cannot simultaneously distinguish between gene loss and introgression, rule out homoplasy due to independent mutations, and connect distinct introgression events to a common subgenome (Buggs et al. 2009; Wang et al. 2009; Salmon et al. 2010). Although our phylogeny is well supported, a large number of sites display homoplasy (fig. 6). We identified 48 genomic clusters containing at least two regions consistent with introgression for a particular subgenome, and with few or no markers supportive of the phylogenetic position of that subgenome. There are at least two reasons why these 48 clusters are likely to represent true introgression events. First, all of them convert an *F. iinumae*-like subgenome (Bi, B1, or B2) to be more *F. vesca*-like, whereas none of them converts subgenome Av to be more *F. iinumae*-like, a highly nonrandom pattern. Second, instances of introgression homoplasy are much more common than outgroup homoplasy, even though both should occur at a similar rate if they are only due to mutation, sequencing errors, or incomplete linage sorting (Green et al. 2010). These 48 clusters represent a nontrivial portion of the haploid genome (7%), and because our conservative method has missed any introgression events that do not span two marker-containing regions, the true proportion of the genome showing introgression is likely even higher. Our whole-genome sequencing of the parents indicates that mapped *F. vesca*-like reads are at least as common as mapped *F. iinumae*-like reads in approximately 30% of the genome outside of our designated introgression regions (fig. 8). Because *F. vesca*-like reads have a higher probability of mapping to Fvb, we are reluctant to use this ratio alone to predict introgression. Nevertheless, genomic sections outside of the introgression clusters but showing an exceptionally high proportion of *F. vesca*-like reads (fig. 8) may represent additional introgression events. Note that the failure of some *F. iinumae*-like sequence to map to Fvb is unlikely to be the cause of the unidirectional introgression pattern, as all informative markers had nonzero coverage in *F. iinumae* by definition.

Introgression could have occurred either before or after polyploidization. If it occurred before then, it would represent ancestral homoploid hybridization events among the diploid ancestors. Specifically, the *F. iinumae*-like diploids contributing to subgenomes B1, B2, and Bi would have had a small amount of *F. vesca* ancestry due to interspecies crossing followed by backcrossing to *F. iinumae*, without a change in chromosome number, prior to the subsequent allopolyploidization event. Because subgenomes B1, B2, and Bi do not form a clade to the exclusion of *F. iinumae*, this scenario requires at least two unrelated homoploid hybrid diploid ancestors. Fossil and biogeographical evidence place the octoploid ancestor in Beringia during the Pleistocene (Liston et al. 2014), and thus the extent of the original hybrid zone and the degree of homoploid introgression may not be reflected in any extant diploid population. Alternatively, introgression could have happened after polyploidization through homeologous exchange. Homeologous gene conversion, the nonreciprocal replacement of an allele with a template sequence from another subgenome, generally happens over short (<1 kb) genomic scales, but has been reported on scales greater than 1 Mb (Jacquemin et al. 2011). Reciprocal homeologous recombination, while not initially eliminating any sequence, will lead to the loss of alleles in progeny that do not inherit both recombinant chromosomes. Reciprocal homeologous recombination can cause extensive change that can rapidly destabilize the genome, which may be why this process is generally suppressed through diploidization in evolutionary successful polyploids (Gaeta and Pires 2010). Many introgression clusters are shared between the two octoploid species (fig. 4; table 2), so if they occurred after polyploidization, then they must have occurred prior to the subsequent speciation of *F. virginiana* and *F. chiloensis*.

The adaptive significance of the observed introgression and its unidirectionality is unknown. Although it is known that the degree of introgression can vary among polyploid subgenomes (Page et al. 2013; Chalhoub et al. 2014), the extreme bias observed here is remarkable and unexpected. If it occurred before polyploidization, and by chance the four diploid ancestors happened to include three homoploid hybrids (*F. iinumae × F. vesca*) and one *F. vesca* individual, natural selection may not have played a role. On the other hand, if such homoploid hybrids were rare, it is unlikely that all three *F. iinumae*-like subgenomes would have hybrid ancestry unless this somehow facilitated polyploid hybridization and the subsequent success of the octoploids. If introgression took place among subgenomes after polyploidization, the unidirectional pattern was likely to have been driven by some consistent evolutionary process, presumably strong selection. Although homeologous exchange can occur preferentially in one direction for mechanistic rather than adaptive reasons (as in Lange et al. 2011), this explanation seems unlikely because the genomes of *F. vesca* and *F. iinumae* are quite similar in length, GC content, and other metrics (Hirakawa et al. 2014). Regardless of when introgression occurred, we can envision two major adaptive hypotheses. First, *F. vesca* sequence per se might have higher fitness. Because *F. vesca* is the cytoplasm donor, there could have been selection for Av subgenome sequence to promote cytonuclear accommodation (Gong et al. 2012), or *F. vesca*-like genes may have been favored in the ecological conditions experienced by the octoploids. We cannot reject this hypothesis, but we note that introgression clusters mostly do not overlap among subgenomes (fig. 4), even though this would be expected if a small number of specific *F. iinumae*-like genes are deleterious in *F. vesca*-like cytoplasm or the ecological habitat of the polyploids. Second, there may have been selection to make the *F. iinumae*-like subgenomes, especially B1 and B2, more divergent from each other, in order to minimize polysomic inheritance which is often associated with lower fitness (Le Comber et al. 2010; Feldman and Levy 2012). It is noteworthy that the *F. iinumae*-like subgenomes typically display disomic inheritance and form independent clades in our phylogenies, despite a recent common origin, particularly for B1 and B2. This puzzling result may be explained by introgression causing rapid divergence among the subgenomes, and it is plausible that the adaptive value of disomic inheritance was in fact a selective advantage favoring introgression.

Genomic rearrangement in polyploids can be extensive (Lim et al. 2004; Udall et al. 2005; Chen and Ni 2006). Transpositions and translocations occurring immediately after polyploidization caused by “genomic shock” have received a great deal of attention (Lim et al. 2004; Chen and Ni 2006), but many rearrangements may also occur in diploid ancestors which are then brought together in allopolyploidy. We observed 139 interchromosome rearrangements in our linkage maps (supplementary table S4, Supplementary Material online; fig. 4). Most of these show direct contradictions, with linkage groups pertaining to different haploid chromosomes containing LG SNPs from the same targeted region in Fvb, and therefore they cannot be explained simply as assembly errors in our reference genome. Our results add to previous observations of interchromosome rearrangements in *F. virginiana* ssp. *virginiana* detected with SSR markers (Spigler et al. 2010). Targeted regions showing rearrangements have higher and more variable depth, relative to diploids, than the rest of the genome, suggesting that homologous sequence is found in multiple locations (fig. 7*B*). In other words, many of these observations may represent a “copy-and-paste” rather than a “cut-and-paste” mechanism, consistent with the activity of Class I or Class II, Subclass 2 transposable elements (Wicker et al. 2007). Of the 110 targeted regions showing rearrangements, 22% contain LG SNPs mapping to linkage groups pertaining to at least two additional Fvb pseudochromosomes, implying repeatedly duplicated segments. After excluding regions that may be duplicated throughout *Fragaria* or just in *F. vesca* ssp. *bracteata* (nontargeted and low octoploid:diploid depth ratio), most rearrangements occur in subgenomes B1, B2, or Bi, reflecting their greater phylogenetic distance from Fvb. Thus, many of the differences between subgenomes are likely to be interchromosome rearrangements that occurred in the diploid ancestors as they diverged from each other. However, those that do occur in subgenome Av must have happened in the relatively short time since this subgenome diverged from its diploid *F. vesca*-like ancestor, suggesting that postpolyploidization rearrangements may also be important, consistent with the common observation of increased transposition in interspecific hybrids (Fontdevila 2005; Chen and Ni 2006). Because our targeted capture approach ignores the majority of the genome, and rearrangements are usually quite small and do not span between targeted regions, there are likely to be many more small rearrangements that were undetected. Our results demonstrate that translocation and/or transposition is a major contributor to differentiation between homeologous chromosomes and is consistent with the hypothesis that transposable elements are important drivers of plant evolution (Lisch 2013). Putative intrachromosome rearrangements are also apparent as changes in LG SNP order within linkage groups relative to Fvb. We did not attempt to quantify intrachromosome translocations because in many cases LG SNP order within linkage groups has low statistical support and would be affected by genotyping errors. However, one very clear inversion stands out, comprising more than half of LG II_B1 in all four octoploid parents (fig. 4). Our data are consistent with this being the same inversion previously identified in octoploid *F. × ananassa* (van Dijk et al. 2014).

Taken together, our results clarify previous observations of cytological and inheritance patterns in *Fragaria*. Although inheritance in octoploid *Fragaria* is primarily disomic, studies of multivalent pairings in hybrids have suggested numerous conflicting cytological models (Ichijima 1926; Yarnell 1931; East 1934; Federova 1946; Senanayake and Bringhurst 1967; Bringhurst 1990). Bringhurst (1990) synthesized the available evidence and proposed a strawberry genomic formula of AAA′A′BBB′B′, subsequently supported by single-gene phylogenies (Rousseau-Gueutin et al. 2009). Our work suggests instead a pattern of AABBB′B′B″B″, similar to the older AABBBBCC model (Federova 1946), with C (Bi) being more similar to B (B1 and B2) than A (Av). Previous *F. vesca* × *F. virginiana* crosses also support a single *F. vesca*-like subgenome, namely, Ichijima (1926) found 7 bivalent and 21 univalent chromosomes, whereas East (1934) found a hybrid in which only a single *F. virginiana* subgenome had been retained. Furthermore, Nathewet et al. (2010) found that most octoploid chromosomes had morphology resembling *F. iinumae*. However, our observation of widespread introgression means that no one pattern holds across all portions of the genome. Indeed, the nuclear gene *DHAR* used by Rousseau-Gueutin et al. (2009) for phylogenetics occurs at Fvb7_16,080,000, just 6 kb upstream from a 386-kb introgression cluster (table 2), and likely encompassed by the introgression event, as the nearest marker showing phylogenetic support is more than 100 kb farther upstream. Thus, although polyphyly of allopolyploids is known (Chalhoub et al. 2014) and cannot be ruled out as an explanation for discrepant results, the data are consistent with a monophyletic octoploid clade but with a phylogenetic signal that varies across the genome. As cytological pairings can be determined by a small number of specific genes (Sears 1976; Vega and Feldman 1998), it is perhaps not surprising to see poor concordance among cytology studies, single-gene phylogenies, and genome-wide phylogenies in many polyploids (Hancock 2012). Indeed, the most appropriate genomic formula for a polyploid may vary depending on whether its intent is to reflect evolutionary history, overall sequence divergence, or extant segregation patterns.

Our new *Fragaria* genome assembly, Fvb, differs from the FvH4 assembly by more than 100 rearrangements (fig. 2). Some of these could represent real translocations and some could represent assembly errors in FvH4. We suspect that a majority represent assembly errors for four reasons. First, we find no inconsistencies among the three *F. vesca* ssp. *bracteata* maps, but if genome rearrangement were so rampant as to have caused all the rearrangements we see, we would expect to see at least one between *F. vesca* ssp. *bracteata* maps from different geographic locations. Second, most rearrangements were consistent with an entire scaffold being misplaced, which is a likely type of methodological error. Third, discrepancies between the octoploids and Fvb rarely support the FvH4 assembly instead, but rather are usually different from both assemblies (supplementary table S4, Supplementary Material online). Fourth, Fvb shows much higher synteny with the *Prunus* genome than FvH4 does (fig. 3). Nearly a third of putative translocations between FvH4 and *Prunus* are eliminated in Fvb, suggesting they are likely assembly errors in FvH4 rather than true genomic rearrangements. Our results underscore the caveat that genome assemblies are imperfect and genomic rearrangements will appear to be more common than they really are due to errors (Bhutkar et al. 2006). For example, the observation that FvH4 shows more genomic rearrangements than other eudicots including *Prunus*, *Coffea*, and *Vitis* (Illa et al. 2011; Denoeud et al. 2014) could be entirely due to assembly errors rather than biological reality. Even if some rearrangements are real differences between *F. vesca* subspecies, the closest diploid relative of the Av subgenome in the octoploids (including the cultivated *F.* × *ananassa*) is *F. vesca* ssp. *bracteata* (Njuguna et al. 2013), and therefore our Fvb assembly will be a useful resource for all strawberry geneticists.

Our approach to genome assembly and polyploid phylogenetics, based on targeted capture and dense linkage maps, will likely be fruitful when applied to other taxa. Several features of our success with this study are particularly noteworthy. First, the Fvb-s map was generated from a single selfed individual, contradicting the common assumption that useful linkage maps can only be generated by crossing distantly related parents. Second, our targeted capture probes were designed to target polymorphisms in Fvb-m and Fvb-p (Tennessen et al. 2013), yet they yielded many useful polymorphisms in Fvb-s and the octoploids, indicating that for sufficiently heterozygous individuals, there is no need to identify polymorphisms in advance. Third, our success with calling segregating SNPs in polyploids suggests that many of the challenges in polyploid genetics (Dufresne et al. 2014) are ameliorated when sequencing coverage is high enough such that variants can be accurately identified even with relatively simple algorithms. Fourth, our novel POLiMAPS method of identifying phylogenetic markers that share short sequencing reads with known linkage map markers will allow otherwise intractable polyploid genomes to be dissected (Hirsch and Buell 2013). Testing for the major patterns described here in other taxa will illuminate the complex evolutionary processes associated with whole-genome duplications.

## Supplementary Material

Supplementary data set S1 and tables S1–S4 are available at *Genome Biology and Evolution* online (http://www.gbe.oxfordjournals.org/).

Supplementary Data
