# Supplementary material for: Evolutionary Origins and Dynamics of Octoploid Strawberry Subgenomes Revealed by Dense Targeted Capture Linkage Maps
Source: Genome Biol Evol. 2014 Dec 4;6(12):3295–313. doi: 10.1093/gbe/evu261 (PMC4986458; doi:10.1093/gbe/evu261)
Supplement: Supplementary Data [file supp_6_12_3295__index.html]

Evolutionary Origins and Dynamics of Octoploid Strawberry Subgenomes Revealed by Dense Targeted Capture Linkage Maps — Supplementary Data 

# Evolutionary Origins and Dynamics of Octoploid Strawberry Subgenomes Revealed by Dense Targeted Capture Linkage Maps

## Supplementary Data

files

**Files in this Data Supplement:**

- Supplementary Data - doc file
- Supplementary Data - txt file
- Supplementary Data - docx file
- Supplementary Data - doc file
- Supplementary Data - doc file
- Supplementary Data - doc file
