# Supplementary material for: Evolutionary Origins and Dynamics of Octoploid Strawberry Subgenomes Revealed by Dense Targeted Capture Linkage Maps
Source: Genome Biol Evol. 2014 Dec 4;6(12):3295–313. doi: 10.1093/gbe/evu261 (PMC4986458; doi:10.1093/gbe/evu261)
Supplement: Supplementary Data [file supp_evu261_Table_S4.doc]

**Table S4.** Regions showing interchromosome rearrangements.

| **Fvb Chrom^a^** | **Fvb Sites^b^** | **LG SNPs^c^** | **Alt LG Count^d^** | **Alt LGs^e^** | **FvH4 Chrom^f^** | **Supportive LGs^g^** |
| --- | --- | --- | --- | --- | --- | --- |
| 1 | 1653924-1654062 | 12 | 1 | Fvirg-V-B1, Fvirg-V-B2, Fvirg-V-Bi, Fchil-V-B2, Fchil-V-Bi | 1 | Fvb-m,Fvb-s |
| 1 | 3458699 | 1 | 1 | Fvirg-III-Bi | 1 | na |
| 1 | 3809587 | 1 | 1 | Fvirg-VI-B2 | 1 | Fvirg-I-Av, |
| 1 | 4675461-4675552 | 2 | 1 | Fvirg-II-B1 | 1 | Fchil-I-B1,Fvb-s |
| 1 | 8067571 | 1 | 1 | Fchil-III-Bi | 0 | Fchil-I-B1,Fchil-I-Av,Fvirg-I-B1,Fvb-p |
| 1 | 19185159 | 1 | 1 | Fanan-II | 7 | na |
| 1 | 20518911 | 1 | 1 | Fchil-IV-Av | 1 | Fvb-m,Fvb-p,Fvb-s |
| 1 | 20672556 | 1 | 1 | Fchil-V-B2 | 1 | Fvb-m,Fvb-p |
| 2 | 1174808 | 1 | 1 | Fvirg-VI | 0 | Fchil-II-B2,Fvirg-II-B1,Fvirg-II-Av,Fvb-m,Fvb-p,Fvb-s |
| 2 | 1330120 | 1 | 1 | Fanan-VI | 0 | na |
| 2 | 1979977 | 1 | 1 | Fanan-VII | 0 | na |
| 2 | 2013920 | 1 | 1 | Fanan-VI | 0 | Fvb-p,Fvb-s |
| 2 | 4921903 | 1 | 1 | Fchil-III-B1 | 0 | Fvirg-II-Av,Fvirg-II-B2,Fvb-p |
| 2 | 6967205 | 1 | 1 | Fanan-III | 2 | na |
| 2 | 7636477-7636739 | 2 | 2 | Fchil-III-Bi, Fchil-VII-Bi | 5 | Fchil-II-B1,Fvb-p |
| 2 | 8030807 | 1 | 1 | Fchil-VI-Bi | 2 | Fchil-II-Av,Fvb-s |
| 2 | 9170267-9170295 | 2 | 2 | Fchil-V-B1, Fvirg-III-B1 | 2 | Fvirg-II-B1,Fvirg-II-Av,Fvb-m |
| 2 | 9366770 | 1 | 1 | Fvirg-VI-B2 | 2 | Fvirg-II-Bi,Fvb-p,Fvb-s |
| 2 | 13244763-13244785 | 2 | 2 | Fchil-V-Av, Fchil-VI-Av | 2 | Fvirg-II-Av,Fvb-m |
| 2 | 15176462-15176613 | 4 | 3 | Fchil-I-Bi, Fvirg-IV-B1, Fvirg-IV-B2, Fvirg-VII-Bi | 2 | Fchil-II-B1 |
| 2 | 15307665 | 1 | 1 | Fvirg-IV-Bi | 2 | Fvirg-II-B2,Fvb-m |
| 2 | 18147570 | 1 | 1 | Fchil-V-B2 | 2 | na |
| 2 | 18269975-18269982 | 2 | 2 | Fvirg-VI-Av, Fvirg-V-Bi | 2 | na |
| 2 | 20616467 | 1 | 1 | Fvirg-III-B1 | 2 | Fvirg-II-Av,Fchil-II-B1,Fvb-p |
| 2 | 21606141 | 1 | 1 | Fchil-IV-B2 | 2 | Fchil-II-B2,Fchil-II-B1,Fvirg-II-B2,Fchil-II-Bi,Fvb-m,Fvb-s |
| 2 | 21962235-21962342 | 2 | 1 | Fchil-V-B1 | 0 | Fchil-II-B2,Fchil-II-Bi,Fvb-m,Fvb-p |
| 2 | 24511560-24511640 | 3 | 1 | Fvirg-V-B1, Fchil-V-B2, Fchil-V-Bi | 2 | Fvirg-II-Av,Fvb-p |
| 2 | 25412494 | 1 | 1 | Fanan-I | 2 | na |
| 2 | 25640422 | 1 | 1 | Fvirg-VII-Av | 2 | na |
| 2 | 25644728 | 1 | 1 | Fchil-IV-B2 | 2 | na |
| 2 | 26148374 | 1 | 1 | Fvirg-V-B1 | 2 | Fvb-m,Fvb-p |
| 2 | 28091651 | 1 | 1 | Fchil-III-Bi | 2 | Fchil-II-Av,Fvirg-II-Av,Fvirg-II-Bi,Fchil-II-B1,Fvb-p |
| 2 | 28561409 | 1 | 1 | Fvirg-VI-B1 | 2 | Fvb-p |
| 3 | 1470682 | 1 | 1 | Fanan-V | 3 | na |
| 3 | 3950235-3950306 | 3 | 3 | Fchil-II-B2, Fvirg-V-Bi, Fvirg-VI-B2 | 3 | Fchil-III-B2 |
| 3 | 4499291-4499488 | 6 | 3 | Fchil-II-B2, Fvirg-IV-Av, Fvirg-IV-B1, Fvirg-IV-B2, Fchil-V-B2 | 3 | Fvirg-III-Av,Fchil-III-Av |
| 3 | 6815542 | 1 | 1 | Fchil-I-B2 | 3 | Fchil-III-B1,Fvirg-III-Av,Fchil-III-Bi,Fvirg-III-Bi,Fchil-III-Av,Fvb-m |
| 3 | 7454977-7455055 | 2 | 1 | Fvirg-IV-B1, Fchil-IV-B2 | 3 | na |
| 3 | 13036170-13036271 | 3 | 1 | Fvirg-II-B1, Fchil-II-Av | 3 | Fvirg-III-B2 |
| 3 | 18062148 | 1 | 1 | Fchil-IV-Av | 3 | na |
| 3 | 21822739-21822743 | 2 | 2 | Fchil-VI-B2, Fchil-VII-Bi | 2 | Fvirg-III-Av,Fchil-III-Bi |
| 3 | 23430678-23430842 | 2 | 2 | Fchil-IV-Av, Fchil-VII-Bi | 3 | na |
| 3 | 24759316-24759489 | 6 | 3 | Fvirg-II-Av, Fvirg-VII-Av, Fchil-VI-B2 | 3 | Fchil-III-Av |
| 3 | 25013920 | 1 | 1 | Fchil-VI-Bi | 3 | na |
| 3 | 32586344-32586439 | 3 | 2 | Fvirg-II-Av, Fchil-II-B1, Fchil-V-B1 | 3 | Fvirg-III-Av,Fvirg-III-Bi,Fvb-m |
| 4 | 306757-306783 | 2 | 1 | Fvirg-III-Bi | 4 | Fvb-m |
| 4 | 1357507-1357643 | 3 | 3 | Fchil-III-B1, Fchil-VI-Bi, Fchil-VII-Bi | 4 | Fvb-p |
| 4 | 3872563 | 1 | 1 | Fvirg-III-Bi | 4 | Fchil-IV-Av,Fvb-m,Fvb-p |
| 4 | 7062388 | 12 | 4 | Fvirg-II-B1, Fvirg-III-B1, Fvirg-VII-Bi, Fchil-II-B1, Fchil-III-B1, Fchil-VI-Av, Fchil-VI-B2, Fchil-VII-Av | 4 | Fvirg-IV-Av,Fchil-IV-Av |
| 4 | 8454076-8454143 | 2 | 1 | Fchil-III-B1, Fchil-III-Bi | 4 | na |
| 4 | 8762860-8762930 | 3 | 3 | Fchil-I-B2, Fchil-II-B1, Fchil-V-B1 | 4 | na |
| 4 | 9056156 | 1 | 1 | Fvirg-II-Bi | 4 | na |
| 4 | 13535603 | 1 | 1 | Fchil-VI-B2 | 4 | Fvirg-IV-Av,Fchil-IV-Av,Fvb-m |
| 4 | 15435498 | 1 | 1 | Fanan-VII | 4 | na |
| 4 | 18212198 | 2 | 1 | Fvirg-I-Av, Fchil-I-Av | 3 | Fchil-IV-Av,Fchil-IV-B2,Fchil-IV-B1,Fchil-IV-Bi, |
| 4 | 19996615 | 1 | 1 | Fvirg-VI-B1 | 4 | Fvb-m |
| 4 | 19999032 | 1 | 1 | Fchil-I-B1 | 4 | Fvirg-IV-B2,Fvb-p |
| 4 | 20095533-20095629 | 4 | 2 | Fvirg-III-Av, Fvirg-V-Bi, Fchil-III-Av | 4 | na |
| 4 | 20829973 | 1 | 1 | Fchil-II-B2 | 4 | Fvb-p |
| 4 | 21242117 | 1 | 1 | Fanan-III | 4 | na |
| 4 | 23324441-23324664 | 2 | 2 | Fvirg-III-B2, Fvirg-V-Bi | 4 | Fvirg-IV-Av,Fchil-IV-Av,Fchil-IV-B1,Fvirg-IV-B1,Fvb-m |
| 4 | 28346486 | 1 | 1 | Fchil-VI-B1 | 4 | Fvb-p |
| 5 | 5896868 | 1 | 1 | Fchil-IV-Bi | 5 | na |
| 5 | 6225764-6226000* | 5 | 1 | Fvirg-III-Av, Fvirg-III-B1, Fchil-III-Av, Fchil-III-B1 Fchil-III-Bi | 0 | Fvb-m |
| 5 | 6236405* | 1 | 1 | Fvirg-III-B2 | 0 | Fvb-m |
| 5 | 8066261-8066287 | 2 | 2 | Fchil-III-Bi, Fchil-IV-Bi | 5 | na |
| 5 | 10590221-10590248 | 2 | 2 | Fvirg-II-B1, Fchil-IV-B1 | 5 | Fvirg-V-Av |
| 5 | 12302247 | 1 | 1 | Fchil-VI-B1 | 5 | Fvb-p |
| 5 | 17910644-17910750 | 2 | 2 | Fvirg-III-Bi, Fchil-VI-Av | 5 | na |
| 5 | 21019145-21019155 | 2 | 1 | Fchil-III-B1 | 5 | Fvb-p,Fvb-s |
| 5 | 21670127-21670234 | 2 | 2 | Fvirg-II-Bi, Fvirg-VII-B2 | 5 | Fvirg-V-Bi,Fvb-p |
| 5 | 22998767* | 1 | 1 | Fanan-III | 3 | na |
| 5 | 23051015* | 1 | 1 | Fanan-III | 3 | na |
| 5 | 23111458* | 1 | 1 | Fchil-III-Av | 3 | na |
| 5 | 23156629-23156710* | 2 | 1 | Fvirg-III-Av, Fchil-III-B1 | 3 | na |
| 5 | 27371279-27371412 | 3 | 2 | Fchil-III-Av, Fchil-III-B1, Fchil-VI-B2 | 5 | na |
| 5 | 29086580 | 1 | 1 | Fchil-II-Av | 5 | Fvirg-V-Bi,Fvb-m |
| 5 | 29225693 | 1 | 1 | Fchil-VI-B1 | 6 | Fvirg-V-Bi,Fvb-p |
| 6 | 1239049 | 1 | 1 | Fchil-III-B2 | 6 | na |
| 6 | 5110827-5110942* | 2 | 1 | Fvirg-II-Bi, Fchil-II-B2 | 6 | Fvirg-VI-Bi,Fchil-VI-B2,Fvirg-VI-B2,Fchil-VI-B1,Fchil-VI-Bi,Fvirg-VI-Av,Fchil-VI-Av,Fvirg-VI-B1,Fvb-m,Fvb-p |
| 6 | 5139939* | 1 | 1 | Fvirg-II-Bi | 6 | Fvirg-VI-Bi,Fchil-VI-B2,Fvirg-VI-B2,Fchil-VI-B1,Fchil-VI-Bi,Fvirg-VI-Av,Fchil-VI-Av,Fvirg-VI-B1,Fvb-m,Fvb-p |
| 6 | 6192706 | 1 | 1 | Fanan-III | 6 | na |
| 6 | 11252363-11252504 | 4 | 3 | Fvirg-I-B2, Fvirg-V-Bi, Fchil-II-B1, Fchil-V-Bi | 6 | Fvirg-VI-Av,Fvb-p |
| 6 | 12507936-12507999 | 2 | 1 | Fvirg-V-B1 | 6 | Fchil-VI-Av |
| 6 | 15816432 | 1 | 1 | Fchil-II-Bi | 6 | Fchil-VI-Bi,Fvb-m,Fvb-p,Fvb-s |
| 6 | 21196974 | 1 | 1 | Fchil-II-Bi | 6 | Fvb-p |
| 6 | 22398274-22398407 | 2 | 2 | Fchil-II-B1, Fvirg-III-B2 | 6 | Fchil-VI-B1,Fchil-VI-Av,Fvirg-VI-Av,Fvirg-VI-B1,Fvb-p |
| 6 | 22959153-22959269 | 3 | 2 | Fvirg-III-B1, Fchil-VII-Bi | 6 | Fchil-VI-B1,Fvirg-VI-Av,Fvb-p |
| 6 | 23077697 | 1 | 1 | Fchil-IV-Av | 6 | Fchil-VI-Av,Fvb-m,Fvb-p,Fvb-s |
| 6 | 23203640 | 1 | 1 | Fchil-II-B1 | 6 | Fvb-s |
| 6 | 25923488 | 1 | 1 | Fvirg-III-B1 | 4 | na |
| 6 | 33043819 | 1 | 1 | Fchil-I-Bi | 6 | Fchil-VI-Av,Fvirg-VI-Av,Fvirg-VI-B1,Fvb-m,Fvb-p,Fvb-s |
| 6 | 35618840 | 1 | 1 | Fchil-II-Av | 6 | Fvirg-VI-B2,Fvirg-VI-Bi,Fchil-VI-B2, |
| 6 | 36277398 | 1 | 1 | Fvirg-III-Bi | 7 | Fvb-m |
| 6 | 36933335* | 1 | 1 | Fanan-II | 6 | Fchil-VI-B1,Fvirg-VI-B2,Fchil-VI-Bi,Fchil-VI-Av,Fvirg-VI-Bi,Fchil-VI-B2,Fvirg-VI-B1,Fvb-m |
| 6 | 36978607* | 1 | 1 | Fchil-II-B2 | 6 | Fchil-VI-B1,Fvirg-VI-B2,Fchil-VI-Bi,Fchil-VI-Av,Fvirg-VI-Bi,Fchil-VI-B2,Fvirg-VI-B1,Fvb-m |
| 6 | 37337398 | 1 | 1 | Fchil-I-Bi | 6 | Fchil-VI-Bi |
| 6 | 37868446 | 1 | 1 | Fanan-IV | 6 | na |
| 6 | 38303994 | 1 | 1 | Fvirg-V-B1 | 6 | Fvb-m,Fvb-p,Fvb-s |
| 7 | 3862355 | 1 | 1 | Fchil-I-B2 | 7 | Fchil-VII-B2,Fchil-VII-Bi,Fvb-m,Fvb-p |
| 7 | 5511085-5511158 | 2 | 1 | Fchil-VI-Bi | 7 | na |
| 7 | 6265866 | 1 | 1 | Fchil-I-B1 | 7 | na |
| 7 | 6936966 | 1 | 1 | Fchil-I-B1 | 7 | Fchil-VII-Av,Fvb-m,Fvb-p |
| 7 | 10043862 | 1 | 1 | Fanan-III | 3 | na |
| 7 | 10227088 | 1 | 1 | Fanan-III | 3 | na |
| 7 | 12279493 | 1 | 1 | Fvirg-VI-B1 | 7 | Fvirg-VII-B1,Fvb-m |
| 7 | 14472225 | 1 | 1 | Fchil-I-Av | 7 | na |
| 7 | 15488112-15488292 | 5 | 1 | Fvirg-VI-Av, Fvirg-VI-B1, Fvirg-VI-Bi, Fchil-VI-B1, Fchil-VI-Bi | 7 | Fchil-VII-B1,Fvirg-VII-B2 |
| 7 | 21951752-21951828 | 3 | 3 | Fvirg-II-Bi, Fvirg-V-Bi, Fvirg-VI-B1 | 7 | Fchil-VII-B2,Fvb-p |
| 7 | 22083323 | 1 | 1 | Fchil-III-Av | 7 | Fchil-VII-B2 |

*Regions that putatively share the same interchromosome rearrangement with an adjacent region. There are four such region-spanning rearrangements.

^a^Pseudochromosome in Fvb reference genome

^b^Physical position in Fvb reference genome

^c^Number of LG SNPs in this region

^d^Number of independent interchromosome rearrangements in this region

^e^Linkage groups supporting rearrangements. Interchromosome rearrangements are defined by markers with linkage group number (roman numeral) that does not match the Fvb pseudochromosome. Fchil = *F. chiloensis*; Fvirg = *F. virginiana*; Fanan = *Fragaria* × *ananassa*

^f^Pseudochromosome in FvH4 reference genome

^g^Linkage groups that support the Fvb position of this region. Fchil = *F. chiloensis*; Fvirg = *F. virginiana*; Fvb = *F. vesca* ssp. *bracteata*; na = no maps have markers supporting the reference genome in this position (scaffold was placed in assembly based on markers elsewhere on the scaffold or by defaulting to FvH4 position).
