# Supplementary material for: Evolutionary Origins and Dynamics of Octoploid Strawberry Subgenomes Revealed by Dense Targeted Capture Linkage Maps
Source: Genome Biol Evol. 2014 Dec 4;6(12):3295–313. doi: 10.1093/gbe/evu261 (PMC4986458; doi:10.1093/gbe/evu261)
Supplement: Supplementary Data [file supp_evu261_New_Microsoft_Office_Word_Document.docx]

**Supplementary Material**

**Table S1.** Samples examined in this study.

**Table S2.** Previous names of octoploid *Fragaria* linkage groups united with subgenome-based names.

**Table S3.** Scaffold positions in FvH4 and Fvb.

**Table S4.** Regions showing interchromosome rearrangements

**Dataset S1.** R script for creating Figure 4.
