# Supplementary material for: Evolutionary Origins and Dynamics of Octoploid Strawberry Subgenomes Revealed by Dense Targeted Capture Linkage Maps
Source: Genome Biol Evol. 2014 Dec 4;6(12):3295–313. doi: 10.1093/gbe/evu261 (PMC4986458; doi:10.1093/gbe/evu261)
Supplement: Supplementary Data [file supp_evu261_Table_S2.doc]

**Table S2.** Previous names of octoploid *Fragaria* linkage groups united with phylogenetic subgenome-based names.

| ***F. virginiana*** | | ***F. chiloensis*** | |
| --- | --- | --- | --- |
| **Spigler et al. 2010** | **This study** | **Goldberg et al. 2010** | **This study** |
| Fvirg-I-A-m | Fvirg-I-Av-m | Fchil-VI-A-m^a^ | Fchil-VI-Av-m |
| Fvirg-I-B-m | Fvirg-I-B1-m | Fchil-VI-B-m | Fchil-VI-B1-m |
| Fvirg-I-C-m | Fvirg-I-B2-m | Fchil-VI-C-m | Fchil-VI-B2-m |
| Fvirg-I-D-m | Fvirg-I-Bi-m | Fchil-VI-D-m | Fchil-VI-Bi-m |
| Fvirg-II-A-m | Fvirg-II-Av-m | Fchil-VI-A-p | Fchil-VI-Av-p |
| Fvirg-II-B-m | Fvirg-II-Bi-m | Fchil-VI-B-p | Fchil-VI-B1-p |
| Fvirg-II-C-m | Fvirg-II-B2-m | Fchil-VI-C-p | Fchil-VI-B2-p |
| Fvirg-II-D-m | Fvirg-II-B1-m | Fchil-VI-D-p | Fchil-VI-Bi-p |
| Fvirg-III-A-m | Fvirg-III-Bi-m |  |  |
| Fvirg-III-B-m-1-m | Fvirg-III-B2-m |  |  |
| Fvirg-III-C-m | Fvirg-III-B1-m |  |  |
| Fvirg-III-D-m | Fvirg-III-Av-m |  |  |
| Fvirg-IV-A-m | Fvirg-IV-Av-m |  |  |
| Fvirg-IV-B-m | Fvirg-IV-B1-m |  |  |
| Fvirg-IV-C-m | Fvirg-IV-Bi-m |  |  |
| Fvirg-IV-D-m | Fvirg-IV-B2-m |  |  |
| Fvirg-V-A-m | Fvirg-V-B1-m |  |  |
| Fvirg-V-B-m | Fvirg-V-B2-m |  |  |
| Fvirg-V-C-m | Fvirg-V-Av-m |  |  |
| Fvirg-V-D-m-2-m-3-m | Fvirg-V-Bi-m |  |  |
| Fvirg-VI-A-m | Fvirg-VI-Av-m |  |  |
| Fvirg-VI-B-m | Fvirg-VI-B1-m |  |  |
| Fvirg-VI-C-m^b^ | Fvirg-VI-B2-m |  |  |
| Fvirg-VI-D-m-4-m | Fvirg-VI-Bi-m |  |  |
| Fvirg-VII-A-m | Fvirg-VII-B2-m |  |  |
| Fvirg-VII-B-m | Fvirg-VII-Av-m |  |  |
| Fvirg-VII-C-m | Fvirg-VII-B1-m |  |  |
| Fvirg-VII-D-m | Fvirg-VII-Bi-m |  |  |
| Fvirg-I-A-p | Fvirg-I-Av-p |  |  |
| Fvirg-I-B-p | Fvirg-I-B1-p |  |  |
| Fvirg-I-C-p | Fvirg-I-B2-p |  |  |
| Fvirg-I-D-p | Fvirg-I-Bi-p |  |  |
| Fvirg-II-A-p | Fvirg-II-Av-p |  |  |
| Fvirg-II-B-p | Fvirg-II-Bi-p |  |  |
| Fvirg-II-C-p | Fvirg-II-B2-p |  |  |
| Fvirg-II-D-p | Fvirg-II-B1-p |  |  |
| Fvirg-III-A-p | Fvirg-III-Bi-p |  |  |
| Fvirg-III-B-p | Fvirg-III-B2-p |  |  |
| Fvirg-III-C-p | Fvirg-III-B1-p |  |  |
| Fvirg-III-D-p | Fvirg-III-Av-p |  |  |
| Fvirg-IV-A-p | Fvirg-IV-Av-p |  |  |
| Fvirg-IV-B-p | Fvirg-IV-B1-p |  |  |
| Fvirg-IV-C-p | Fvirg-IV-Bi-p |  |  |
| Fvirg-IV-D-p | Fvirg-IV-B2-p |  |  |
| Fvirg-V-A-p | Fvirg-V-B1-p |  |  |
| Fvirg-V-B-p | Fvirg-V-B2-p |  |  |
| Fvirg-V-C-p | Fvirg-V-Av-p |  |  |
| Fvirg-V-D-p | Fvirg-V-Bi-p |  |  |
| Fvirg-VI-A-p-2-p | Fvirg-VI-Av-p |  |  |
| Fvirg-VI-B-p | Fvirg-VI-B1-p |  |  |
| Fvirg-VI-C-p | Fvirg-VI-B2-p |  |  |
| Fvirg-VI-D-p-3-p | Fvirg-VI-Bi-p |  |  |
| Fvirg-VII-A-p | Fvirg-VII-Bi-p |  |  |
| Fvirg-VII-B-p | Fvirg-VII-Av-p |  |  |
| Fvirg-VII-C-p | Fvirg-VII-B1-p |  |  |
| Fvirg-VII-D-p | Fvirg-VII-B2-p |  |  |

^a^*F. chiloensis* sex chromosome

^b^*F. virginiana* sex chromosome
