# Supplementary material for: Evolutionary Origins and Dynamics of Octoploid Strawberry Subgenomes Revealed by Dense Targeted Capture Linkage Maps
Source: Genome Biol Evol. 2014 Dec 4;6(12):3295–313. doi: 10.1093/gbe/evu261 (PMC4986458; doi:10.1093/gbe/evu261)
Supplement: Supplementary Data [file supp_evu261_Table_S3.doc]

**Table S3.** Scaffold positions in FvH4 and Fvb.

| **Scaffold^a^** | **FvH4 Chrom^b^** | **FvH4 Original Range^c^** | **FvH4 Trimmed Range^d^** | **Fvb-s^e^** | **Fvb-m^f^** | **Fvb-p^g^** | **Fvb Chrom^h^** | **Fvb Range^i^** | **Orientation^j^** |
| --- | --- | --- | --- | --- | --- | --- | --- | --- | --- |
| scf0512888 | 0 | 1-66745 | 1-66745 | 0 | 0 | 0 | 0 | 1-66745 | 0 |
| scf0512937 | 0 | 76746-307809 | 76746-307809 | 0 | 0 | 0 | 0 | 76746-307809 | 0 |
| scf0512994 | 0 | 1113779-1189430 | 1113779-1189430 | 0 | 0 | 0 | 0 | 317810-393461 | 0 |
| scf0513038 | 0 | 2168184-2242494 | 2168184-2242494 | 0 | 0 | 0 | 0 | 403462-477772 | 0 |
| scf0513075 | 0 | 3355237-4355947 | 3355237-4355947 | 0 | 0 | 0 | 0 | 487773-1488483 | 0 |
| scf0513133 | 0 | 6851402-7316932 | 6851402-7316932 | 0 | 0 | 0 | 0 | 1498484-1964014 | 0 |
| scf0513151b | 1 | 58501-743000 | 58907-742378 | 21 | 31 | 23 | 1 | 1-683472 | -1 |
| scf0513105 | 1 | 1455377-3331343 | 1455377-3331343 | 63 | 97 | 13 | 1 | 693473-2569439 | -1 |
| scf0512959b | 1 | 5155001-5419653 | 5155001-5419653 | 7 | 16 | 0 | 1 | 2579440-2844092 | -1 |
| scf0513146_5b | 5 | 21658001-21946032 | 21658446-21946031 | 6 | 19 | 0 | 1 | 2854093-3141678 | 1 |
| scf0513146_1 | 1 | 4130632-4819946 | 4136762-4819946 | 6 | 23 | 1 | 1 | 3151679-3834863 | 1 |
| scf0513192 | 1 | 5429654-8865332 | 5429654-8865332 | 45 | 120 | 85 | 1 | 3844864-7280542 | 1 |
| scf0513081 | 0 | 4365948-5638289 | 4365948-5638289 | 19 | 40 | 39 | 1 | 7290543-8562884 | -1 |
| scf0512993 | 1 | 10418797-10825620 | 10418797-10825620 | 0 | 0 | 0 | 1 | 8572885-8979708 | 0 |
| scf0512948 | 1 | 10835621-10906189 | 10835621-10906189 | 0 | 0 | 0 | 1 | 8989709-9060277 | 0 |
| scf0513007 | 1 | 10916190-11034326 | 10916190-11034326 | 0 | 1 | 1 | 1 | 9070278-9188414 | 0 |
| scf0512991b | 6 | 34690001-36036831 | 34690001-36036831 | 25 | 44 | 31 | 1 | 9198415-10545245 | -1 |
| scf0512986 | 1 | 14582915-14689724 | 14582915-14689724 | 4 | 3 | 4 | 1 | 10555246-10662055 | 0 |
| scf0513160_1 | 1 | 14699725-15937868 | 14699725-15937868 | 2 | 26 | 30 | 1 | 10672056-11910199 | 1 |
| scf0513066 | 5 | 13220304-13294433 | 13220304-13294432 | 0 | 3 | 0 | 1 | 11920200-11994328 | -2 |
| scf0513041 | 0 | 2252495-2721037 | 2252495-2721037 | 10 | 13 | 9 | 1 | 12004329-12472871 | -2 |
| scf0513039 | 1 | 17147741-17329064 | 17147741-17329064 | 0 | 10 | 3 | 1 | 12482872-12664195 | 0 |
| scf0513189a | 0 | 11189146-11752000 | 11189146-11751595 | 0 | 5 | 5 | 1 | 12674196-13236645 | 0 |
| scf0513096 | 1 | 9928166-10408796 | 9928166-10408796 | 1 | 0 | 0 | 1 | 13246646-13727276 | 0 |
| scf0513030 | 1 | 11809777-12404014 | 11809777-12404014 | 3 | 1 | 1 | 1 | 13737277-14331514 | 0 |
| scf0512886 | 1 | 12414015-12485203 | 12414015-12485203 | 0 | 0 | 0 | 1 | 14341515-14412703 | 0 |
| scf0513175 | 1 | 12495204-13176591 | 12495204-13176591 | 5 | 0 | 0 | 1 | 14422704-15104091 | 0 |
| scf0513095b | 1 | 13768001-14572914 | 13768150-14572914 | 1 | 11 | 0 | 1 | 15114092-15918856 | 0 |
| scf0513168_1a | 1 | 17339065-19036000 | 17339065-19035928 | 14 | 27 | 18 | 1 | 15928857-17625720 | 0 |
| scf0513056 | 1 | 19948126-20231648 | 19948126-20231648 | 2 | 1 | 1 | 1 | 17635721-17919243 | 0 |
| scf0513076 | 1 | 15947869-16084423 | 15947869-16084423 | 0 | 1 | 0 | 1 | 17929244-18065798 | 0 |
| scf0513021 | 1 | 16094424-16205652 | 16094424-16205652 | 0 | 1 | 0 | 1 | 18075799-18187027 | 0 |
| scf0512958 | 1 | 16215653-16369074 | 16215653-16369074 | 0 | 3 | 1 | 1 | 18197028-18350449 | 0 |
| scf0513051 | 1 | 16379075-16703311 | 16379075-16703311 | 0 | 0 | 3 | 1 | 18360450-18684686 | 0 |
| scf0513048 | 1 | 16713312-17137740 | 16713312-17137740 | 4 | 4 | 4 | 1 | 18694687-19119115 | 0 |
| scf0513170b | 7 | 599501-827000 | 599705-826845 | 1 | 0 | 1 | 1 | 19129116-19356256 | 0 |
| scf0513151c | 1 | 743001-1445376 | 743256-1445376 | 1 | 0 | 0 | 1 | 19366257-20068377 | -1 |
| scf0513125a | 3 | 19509464-19755500 | 19509464-19754903 | 1 | 0 | 0 | 1 | 20078378-20323817 | 0 |
| scf0513059 | 1 | 8875333-9022590 | 8875333-9022590 | 0 | 0 | 0 | 1 | 20333818-20481075 | 0 |
| scf0513103 | 1 | 9032591-9827394 | 9032591-9827394 | 3 | 4 | 4 | 1 | 20491076-21285879 | 0 |
| scf0513046 | 1 | 9837395-9918165 | 9837395-9918165 | 0 | 0 | 0 | 1 | 21295880-21376650 | 0 |
| scf0513033 | 1 | 11044327-11161160 | 11044327-11161160 | 0 | 0 | 0 | 1 | 21386651-21503484 | 0 |
| scf0513035 | 1 | 11171161-11239461 | 11171161-11239461 | 0 | 0 | 0 | 1 | 21513485-21581785 | 0 |
| scf0513176a | 6 | 16117078-16640000 | 16117078-16635179 | 1 | 2 | 1 | 1 | 21591786-22109887 | 0 |
| scf0513120 | 1 | 11249462-11799776 | 11249462-11799776 | 4 | 1 | 1 | 1 | 22119888-22670202 | 0 |
| scf0513141 | 1 | 20241649-20954957 | 20241649-20954957 | 2 | 0 | 0 | 1 | 22680203-23393511 | 0 |
| scf0513189b | 0 | 11752001-11767950 | 11752239-11767950 | 0 | 1 | 0 | 2 | 1-15712 | 0 |
| scf0513112b | 6 | 38722001-39347594 | 38722001-39347594 | 12 | 17 | 1 | 2 | 25713-651306 | 0 |
| scf0513068 | 0 | 2802573-3345236 | 2802573-3345236 | 15 | 20 | 10 | 2 | 661307-1203970 | -2 |
| scf0513119 | 0 | 5648290-6841401 | 5648290-6841401 | 19 | 33 | 31 | 2 | 1213971-2407082 | 0 |
| scf0513131 | 2 | 1-2262726 | 1-2262726 | 74 | 44 | 57 | 2 | 2417083-4679808 | 0 |
| scf0513137 | 0 | 7326933-8406320 | 7326933-8406320 | 6 | 24 | 24 | 2 | 4689809-5769196 | 0 |
| scf0512953 | 2 | 2466318-2530947 | 2466318-2530947 | 0 | 0 | 0 | 2 | 5779197-5843826 | 0 |
| scf0513125c | 3 | 20895001-21597487 | 20895001-21597487 | 1 | 29 | 1 | 2 | 5853827-6556313 | 0 |
| scf0512964 | 2 | 2540948-2900227 | 2540948-2900227 | 7 | 7 | 14 | 2 | 6566314-6925593 | 0 |
| scf0513116 | 2 | 3456100-4095607 | 3456100-4095607 | 10 | 7 | 14 | 2 | 6935594-7575101 | 0 |
| scf0513052 | 5 | 3595758-3984026 | 3595758-3984026 | 0 | 1 | 19 | 2 | 7585102-7973370 | 0 |
| scf0513172 | 2 | 4105608-4338477 | 4105608-4338477 | 8 | 3 | 6 | 2 | 7983371-8216240 | 0 |
| scf0513097 | 2 | 4348478-4975127 | 4348478-4975127 | 12 | 5 | 27 | 2 | 8226241-8852890 | 0 |
| scf0513100 | 2 | 4985128-5694769 | 4985128-5694769 | 11 | 15 | 15 | 2 | 8862891-9572532 | 0 |
| scf0512973 | 2 | 6010075-6373169 | 6010075-6373169 | 3 | 6 | 6 | 2 | 9582533-9945627 | 0 |
| scf0512984 | 2 | 6383170-7303387 | 6383170-7303387 | 20 | 15 | 31 | 2 | 9955628-10875845 | 0 |
| scf0512957 | 2 | 7313388-7507128 | 7313388-7507128 | 0 | 12 | 2 | 2 | 10885846-11079586 | 0 |
| scf0513108 | 2 | 7517129-8211702 | 7517129-8211702 | 0 | 4 | 3 | 2 | 11089587-11784160 | 0 |
| scf0513044c | 7 | 19074301-19080000 | 19074301-19080000 | 0 | 1 | 1 | 2 | 11794161-11799860 | 0 |
| scf0513013 | 2 | 8221703-8459417 | 8221703-8459417 | 0 | 4 | 7 | 2 | 11809861-12047575 | 0 |
| scf0513036 | 2 | 8469418-8545024 | 8469418-8545024 | 0 | 4 | 0 | 2 | 12057576-12133182 | 0 |
| scf0512966 | 2 | 8555025-8665654 | 8555025-8665654 | 0 | 4 | 0 | 2 | 12143183-12253812 | 0 |
| scf0513139_2 | 2 | 8675655-8794998 | 8675655-8794998 | 0 | 0 | 0 | 2 | 12263813-12383156 | 0 |
| scf0513074 | 2 | 8804999-9308931 | 8804999-9308931 | 9 | 16 | 15 | 2 | 12393157-12897089 | 0 |
| scf0513042 | 2 | 9318932-9433883 | 9318932-9433883 | 1 | 0 | 3 | 2 | 12907090-13022041 | 0 |
| scf0512989 | 2 | 2910228-3446099 | 2910228-3446098 | 2 | 25 | 8 | 2 | 13032042-13567912 | 1 |
| scf0513164 | 2 | 9443884-12126193 | 9443884-12126193 | 35 | 98 | 18 | 2 | 13577913-16260222 | 1 |
| scf0513132 | 2 | 12136194-14034273 | 12136194-14034273 | 27 | 71 | 14 | 2 | 16270223-18168302 | 0 |
| scf0513174 | 2 | 14044274-14721811 | 14044274-14721811 | 20 | 24 | 16 | 2 | 18178303-18855840 | -1 |
| scf0513123 | 2 | 14731812-15671829 | 14731812-15671829 | 17 | 36 | 20 | 2 | 18865841-19805858 | 1 |
| scf0513018 | 0 | 1199431-1488070 | 1199431-1488070 | 5 | 10 | 17 | 2 | 19815859-20104498 | -1 |
| scf0513144 | 2 | 15681830-17472695 | 15681830-17472695 | 25 | 67 | 63 | 2 | 20114499-21905364 | 1 |
| scf0513148 | 0 | 9021620-9922368 | 9021620-9922368 | 5 | 23 | 27 | 2 | 21915365-22816113 | 1 |
| scf0512938 | 2 | 2272727-2456317 | 2272727-2456317 | 0 | 11 | 4 | 2 | 22826114-23009704 | 0 |
| scf0513196a | 6 | 13162357-13850000 | 13162357-13850000 | 3 | 9 | 12 | 2 | 23019705-23707348 | 0 |
| scf0512968 | 2 | 17482696-18535496 | 17482696-18535496 | 2 | 22 | 32 | 2 | 23717349-24770149 | -1 |
| scf0512943 | 2 | 18545497-18657365 | 18545497-18657365 | 0 | 0 | 3 | 2 | 24780150-24892018 | 0 |
| scf0513134 | 2 | 18667366-21445916 | 18667366-21445916 | 27 | 82 | 81 | 2 | 24902019-27680569 | -1 |
| scf0513194a | 2 | 21455917-22710000 | 21455917-22710000 | 5 | 26 | 47 | 2 | 27690570-28944653 | -1 |
| scf0513171 | 3 | 1-1497153 | 1-1497153 | 8 | 55 | 30 | 3 | 1-1497153 | -1 |
| scf0513012 | 5 | 1-128260 | 1-128260 | 0 | 4 | 3 | 3 | 1507154-1635413 | 1 |
| scf0513104b | 3 | 2400001-3362024 | 2400001-3362024 | 12 | 31 | 18 | 3 | 1645414-2607437 | -1 |
| scf0513168_1c | 1 | 19533001-19539000 | 19533001-19539000 | 1 | 0 | 2 | 3 | 2617438-2623437 | 0 |
| scf0513104a | 3 | 1507154-2400000 | 1507154-2400000 | 7 | 24 | 23 | 3 | 2633438-3526284 | -1 |
| scf0513017 | 3 | 3737614-4232872 | 3737614-4232872 | 1 | 12 | 6 | 3 | 3536285-4031543 | -1 |
| scf0512992 | 3 | 4242873-4400663 | 4242873-4400663 | 0 | 2 | 1 | 3 | 4041544-4199334 | 0 |
| scf0513082 | 3 | 4410664-5334373 | 4410664-5334373 | 7 | 49 | 31 | 3 | 4209335-5133044 | -1 |
| scf0512967 | 3 | 5344374-5686082 | 5344374-5686082 | 5 | 6 | 2 | 3 | 5143045-5484753 | 0 |
| scf0512940 | 3 | 5696083-5852419 | 5696083-5852419 | 0 | 3 | 1 | 3 | 5494754-5651090 | 0 |
| scf0513091 | 3 | 5862420-6446994 | 5862420-6446994 | 16 | 9 | 4 | 3 | 5661091-6245665 | -1 |
| scf0513173 | 3 | 6456995-8718722 | 6456995-8718722 | 20 | 92 | 23 | 3 | 6255666-8517393 | -1 |
| scf0512971 | 3 | 8728723-8799562 | 8728723-8799562 | 0 | 0 | 0 | 3 | 8527394-8598233 | 0 |
| scf0512941 | 3 | 8809563-8892723 | 8809563-8892723 | 0 | 0 | 0 | 3 | 8608234-8691394 | 0 |
| scf0512970 | 3 | 8902724-9035059 | 8902724-9035059 | 0 | 0 | 0 | 3 | 8701395-8833730 | 0 |
| scf0513170a | 7 | 1-599500 | 1-599163 | 8 | 20 | 0 | 3 | 8843731-9442893 | 1 |
| scf0513086 | 3 | 24831844-25293470 | 24831844-25293470 | 8 | 9 | 0 | 3 | 9452894-9914520 | 0 |
| scf0513142 | 3 | 25303471-25849224 | 25303471-25849224 | 7 | 22 | 0 | 3 | 9924521-10470274 | 1 |
| scf0512947 | 0 | 496711-761616 | 496711-761616 | 3 | 0 | 0 | 3 | 10480275-10745180 | 0 |
| scf0513107 | 3 | 9393134-9799968 | 9393134-9799968 | 2 | 0 | 0 | 3 | 10755181-11162015 | 0 |
| scf0513157 | 3 | 11913824-14764165 | 11913824-14764165 | 18 | 0 | 0 | 3 | 11172016-14022357 | 0 |
| scf0513129 | 3 | 14774166-15194651 | 14774166-15194651 | 0 | 0 | 0 | 3 | 14032358-14452843 | 0 |
| scf0513064 | 3 | 15618969-15859681 | 15618969-15859681 | 0 | 0 | 0 | 3 | 14462844-14703556 | 0 |
| scf0513005 | 3 | 15869682-16337949 | 15869682-16337949 | 0 | 0 | 0 | 3 | 14713557-15181824 | 0 |
| scf0513084 | 3 | 16347950-16441952 | 16347950-16441952 | 0 | 0 | 0 | 3 | 15191825-15285827 | 0 |
| scf0513092 | 3 | 16451953-16802391 | 16451953-16802391 | 0 | 0 | 0 | 3 | 15295828-15646266 | 0 |
| scf0513079 | 3 | 16812392-17057038 | 16812392-17057038 | 0 | 0 | 0 | 3 | 15656267-15900913 | 0 |
| scf0513126 | 3 | 17067039-17575972 | 17067039-17575972 | 0 | 0 | 0 | 3 | 15910914-16419847 | 0 |
| scf0512979 | 3 | 17585973-17832162 | 17585973-17832162 | 0 | 0 | 0 | 3 | 16429848-16676037 | 0 |
| scf0513047 | 3 | 17842163-17934453 | 17842163-17934453 | 0 | 0 | 0 | 3 | 16686038-16778328 | 0 |
| scf0513071 | 3 | 17944454-18182276 | 17944454-18182276 | 0 | 0 | 0 | 3 | 16788329-17026151 | 0 |
| scf0513009 | 3 | 18192277-18649760 | 18192277-18649760 | 0 | 0 | 0 | 3 | 17036152-17493635 | 0 |
| scf0513118 | 3 | 18659761-19499463 | 18659761-19499463 | 0 | 0 | 0 | 3 | 17503636-18343338 | 0 |
| scf0513130 | 3 | 23423533-24821843 | 23423533-24821843 | 15 | 0 | 0 | 3 | 18353339-19751649 | 0 |
| scf0513106b | 5 | 14384001-14787580 | 14386378-14787580 | 1 | 0 | 0 | 3 | 19761650-20162852 | -2 |
| scf0513138 | 0 | 8416321-9011619 | 8416321-9011619 | 6 | 0 | 0 | 3 | 20172853-20768151 | -2 |
| scf0513194b | 2 | 22710001-24538926 | 22710001-24538926 | 3 | 0 | 0 | 3 | 20778152-22607077 | -1 |
| scf0513034 | 0 | 1657225-2158183 | 1657225-2158183 | 1 | 0 | 0 | 3 | 22617078-23118036 | 0 |
| scf0513169 | 3 | 25859225-26398954 | 25859225-26398954 | 0 | 0 | 0 | 3 | 23128037-23667766 | 0 |
| scf0513178_3 | 3 | 26408955-27870399 | 26408955-27870399 | 11 | 0 | 0 | 3 | 23677767-25139211 | 1 |
| scf0513140 | 3 | 21607488-23026956 | 21607488-23026956 | 2 | 0 | 0 | 3 | 25149212-26568680 | 0 |
| scf0513101 | 3 | 23036957-23413532 | 23036957-23413532 | 0 | 0 | 0 | 3 | 26578681-26955256 | 0 |
| scf0513125b | 3 | 19755501-20895000 | 19756368-20895000 | 6 | 0 | 0 | 3 | 26965257-28103889 | -1 |
| scf0513090 | 4 | 7833870-8595096 | 7833870-8595096 | 0 | 0 | 1 | 3 | 28113890-28875116 | 0 |
| scf0512978 | 3 | 28611209-29506294 | 28611209-29506294 | 5 | 0 | 11 | 3 | 28885117-29780202 | 0 |
| scf0513058 | 3 | 29516295-30374733 | 29516295-30374733 | 8 | 0 | 20 | 3 | 29790203-30648641 | 1 |
| scf0512981 | 0 | 1041723-1103778 | 1041723-1103778 | 2 | 0 | 0 | 3 | 30658642-30720697 | 0 |
| scf0513181 | 3 | 30873216-30972797 | 30873216-30972797 | 0 | 0 | 0 | 3 | 30730698-30830279 | 0 |
| scf0513029 | 3 | 30982798-31368128 | 30982798-31368128 | 14 | 0 | 14 | 3 | 30840280-31225610 | -1 |
| scf0513184 | 3 | 31378129-32069524 | 31378129-32069524 | 0 | 0 | 0 | 3 | 31235611-31927006 | 0 |
| scf0512944 | 3 | 30384734-30475470 | 30384734-30475470 | 0 | 0 | 4 | 3 | 31937007-32027743 | 0 |
| scf0513015 | 3 | 27880400-28601208 | 27880400-28601208 | 18 | 8 | 14 | 3 | 32037744-32758552 | 1 |
| scf0512985 | 3 | 30485471-30863215 | 30485471-30863215 | 7 | 12 | 15 | 3 | 32768553-33146297 | 0 |
| scf0513085 | 4 | 1229239-2062315 | 1229239-2062315 | 0 | 32 | 13 | 4 | 1-833077 | 1 |
| scf0512988 | 4 | 216651-328193 | 216651-328193 | 0 | 0 | 0 | 4 | 843078-954620 | 0 |
| scf0513093 | 4 | 338194-1219238 | 338194-1219238 | 0 | 22 | 23 | 4 | 964621-1845665 | 0 |
| scf0513099 | 4 | 2072316-2885883 | 2072316-2885883 | 0 | 27 | 5 | 4 | 1855666-2669233 | 1 |
| scf0512975 | 0 | 932451-1031722 | 932451-1031722 | 0 | 2 | 0 | 4 | 2679234-2778505 | 0 |
| scf0512987 | 4 | 3808591-3995204 | 3808591-3995204 | 0 | 6 | 7 | 4 | 2788506-2975119 | 0 |
| scf0513151a | 1 | 1-58500 | 1-58311 | 0 | 2 | 0 | 4 | 2985120-3043430 | 0 |
| scf0513147 | 4 | 2895884-3798590 | 2895884-3798590 | 0 | 31 | 41 | 4 | 3053431-3956137 | 0 |
| scf0513028 | 0 | 1498071-1647224 | 1498071-1647224 | 0 | 0 | 1 | 4 | 3966138-4115291 | 0 |
| scf0513057 | 2 | 5704770-6000074 | 5704770-6000074 | 0 | 2 | 3 | 4 | 4125292-4420596 | 0 |
| scf0513040 | 6 | 25990763-26193578 | 25990763-26193578 | 0 | 5 | 2 | 4 | 4430597-4633412 | 0 |
| scf0513187b | 5 | 25220001-26212222 | 25220001-26212222 | 0 | 38 | 15 | 4 | 4643413-5635634 | 0 |
| scf0513163 | 4 | 4005205-6879543 | 4005205-6879543 | 0 | 41 | 19 | 4 | 5645635-8519973 | 0 |
| scf0512996 | 4 | 6889544-7277507 | 6889544-7277507 | 0 | 1 | 3 | 4 | 8529974-8917937 | 0 |
| scf0512974 | 4 | 7287508-7353313 | 7287508-7353313 | 0 | 1 | 1 | 4 | 8927938-8993743 | 0 |
| scf0513070 | 4 | 7363314-7823869 | 7363314-7823869 | 0 | 4 | 11 | 4 | 9003744-9464299 | 0 |
| scf0512931 | 4 | 8605097-8696262 | 8605097-8696262 | 0 | 7 | 5 | 4 | 9474300-9565465 | 0 |
| scf0513083 | 4 | 8706263-9515260 | 8706263-9515260 | 0 | 10 | 2 | 4 | 9575466-10384463 | 0 |
| scf0513124_4a | 4 | 9525261-10011000 | 9525261-10011000 | 0 | 17 | 9 | 4 | 10394464-10880203 | 0 |
| scf0513155 | 0 | 9932369-11179145 | 9932369-11179145 | 0 | 4 | 1 | 4 | 10890204-12136980 | 0 |
| scf0513121 | 4 | 10270388-11298316 | 10270388-11298316 | 0 | 6 | 1 | 4 | 12146981-13174909 | 0 |
| scf0512972 | 4 | 11308317-11467659 | 11308317-11467659 | 0 | 5 | 4 | 4 | 13184910-13344252 | 0 |
| scf0513062 | 4 | 11477660-12589597 | 11477660-12589597 | 0 | 28 | 24 | 4 | 13354253-14466190 | 0 |
| scf0513025 | 4 | 23946648-24163290 | 23946648-24163290 | 0 | 8 | 1 | 4 | 14476191-14692833 | 0 |
| scf0513162 | 4 | 15041723-17809065 | 15041723-17809065 | 0 | 72 | 53 | 4 | 14702834-17470176 | -1 |
| scf0513150a | 4 | 12599598-13280000 | 12599598-13280000 | 0 | 1 | 3 | 4 | 17480177-18160579 | 0 |
| scf0513053 | 3 | 15204652-15608968 | 15204652-15608968 | 0 | 2 | 3 | 4 | 18170580-18574896 | -2 |
| scf0513159 | 4 | 17819066-20363311 | 17819066-20363311 | 0 | 53 | 49 | 4 | 18584897-21129142 | 0 |
| scf0513154 | 4 | 21259091-22928186 | 21259091-22928186 | 0 | 30 | 22 | 4 | 21139143-22808238 | 0 |
| scf0513002 | 4 | 20373312-21249090 | 20373312-21249090 | 0 | 31 | 20 | 4 | 22818239-23694017 | -1 |
| scf0512942 | 0 | 317810-486710 | 317810-486710 | 0 | 10 | 3 | 4 | 23704018-23872918 | -2 |
| scf0513004a | 4 | 22938187-23058000 | 22938187-23057662 | 0 | 5 | 4 | 4 | 23882919-24002394 | 0 |
| scf0513160a | 9 | 1-1989362 | 1-1989362 | 0 | 1 | 1 | 4 | 24012395-26001756 | 0 |
| scf0513060 | 4 | 24173291-24288702 | 24173291-24288702 | 0 | 0 | 3 | 4 | 26011757-26127168 | 0 |
| scf0513158_4a | 4 | 24298703-25528000 | 24298703-25527750 | 0 | 40 | 49 | 4 | 26137169-27366216 | 1 |
| scf0513158_4c | 4 | 25582001-26557413 | 25582001-26557413 | 0 | 40 | 33 | 4 | 27376217-28351629 | 1 |
| scf0513158b | 8 | 1-2416151 | 1-2416151 | 4 | 3 | 3 | 4 | 28361630-30777780 | 1 |
| scf0512933 | 4 | 26567414-27214541 | 26567414-27214541 | 10 | 22 | 16 | 4 | 30787781-31434908 | 1 |
| scf0513087a | 5 | 27369861-27431000 | 27369861-27431000 | 0 | 1 | 1 | 4 | 31444909-31506048 | 0 |
| scf0512935 | 4 | 14053387-15031722 | 14053387-15031722 | 7 | 39 | 30 | 4 | 31516049-32494384 | -1 |
| scf0513098 | 5 | 138261-3585757 | 138261-3585757 | 46 | 110 | 130 | 5 | 1-3447497 | 1 |
| scf0513150b | 4 | 13280001-14043386 | 13280001-14043386 | 11 | 27 | 19 | 5 | 3457498-4220883 | 1 |
| scf0513067 | 5 | 5078833-5177396 | 5078833-5177396 | 4 | 1 | 4 | 5 | 4230884-4329447 | 0 |
| scf0513158_5 | 5 | 3994027-5068832 | 3994027-5068832 | 17 | 44 | 40 | 5 | 4339448-5414253 | -1 |
| scf0511962 | 5 | 5833229-5933781 | 5833229-5933781 | 1 | 2 | 9 | 5 | 5424254-5524806 | 0 |
| scf0512956 | 5 | 5187397-5823228 | 5187397-5823228 | 12 | 20 | 20 | 5 | 5534807-6170638 | 1 |
| scf0512960 | 0 | 771617-922450 | 771617-922450 | 0 | 1 | 0 | 5 | 6180639-6331472 | 0 |
| scf0513167 | 5 | 5943782-6219518 | 5943782-6219518 | 2 | 0 | 2 | 5 | 6341473-6617209 | 0 |
| scf0513024 | 5 | 6229519-6740798 | 6229519-6740798 | 4 | 18 | 14 | 5 | 6627210-7138489 | 1 |
| scf0513111 | 5 | 6750799-8562000 | 6750799-8562000 | 21 | 38 | 46 | 5 | 7148490-8959691 | -1 |
| scf0513135c | 5 | 8865001-11121459 | 8865471-11121459 | 18 | 72 | 54 | 5 | 8969692-11225680 | -1 |
| scf0513135a | 5 | 8572001-8844000 | 8572001-8843693 | 1 | 0 | 2 | 5 | 11235681-11507373 | -1 |
| scf0513143 | 5 | 11131460-13210303 | 11131460-13210303 | 18 | 43 | 24 | 5 | 11517374-13596217 | 0 |
| scf0513077 | 5 | 13304434-13371434 | 13304434-13371434 | 0 | 0 | 1 | 5 | 13606218-13673218 | 0 |
| scf0513122 | 5 | 13381435-14067596 | 13381435-14067596 | 4 | 0 | 0 | 5 | 13683219-14369380 | 0 |
| scf0513178_5 | 5 | 15206669-16688241 | 15206669-16688241 | 17 | 23 | 19 | 5 | 14379381-15860953 | 0 |
| scf0512963 | 5 | 27950253-28286745 | 27950253-28286745 | 0 | 2 | 1 | 5 | 15870954-16207446 | 0 |
| scf0513011 | 5 | 28296746-28438568 | 28296746-28438568 | 7 | 1 | 1 | 5 | 16217447-16359269 | 0 |
| scf0513094 | 5 | 17913724-20360920 | 17913724-20360920 | 43 | 62 | 45 | 5 | 16369270-18816466 | -1 |
| scf0513145 | 5 | 17272022-17711282 | 17272022-17711282 | 2 | 2 | 0 | 5 | 18826467-19265727 | 0 |
| scf0513055 | 5 | 17721283-17903723 | 17721283-17903723 | 2 | 0 | 1 | 5 | 19275728-19458168 | 0 |
| scf0513146_5a | 5 | 20370921-21658000 | 20370921-21657932 | 19 | 14 | 28 | 5 | 19468169-20755180 | 1 |
| scf0513166 | 5 | 14797581-15196668 | 14797581-15196668 | 5 | 0 | 4 | 5 | 20765181-21164268 | 0 |
| scf0513139_5 | 5 | 16698242-17262021 | 16698242-17262021 | 3 | 0 | 14 | 5 | 21174269-21738048 | 0 |
| scf0512959a | 1 | 4829947-5155000 | 4829947-5155000 | 4 | 0 | 9 | 5 | 21748049-22073102 | 0 |
| scf0513045 | 0 | 2731038-2792572 | 2731038-2792572 | 0 | 0 | 2 | 5 | 22083103-22144637 | 0 |
| scf0513109 | 5 | 23230628-23604769 | 23230628-23604769 | 3 | 0 | 21 | 5 | 22154638-22528779 | 0 |
| scf0513115 | 3 | 11045986-11903823 | 11045986-11903823 | 0 | 0 | 9 | 5 | 22538780-23396617 | -2 |
| scf0513128 | 5 | 21956033-23220627 | 21956033-23220627 | 21 | 0 | 33 | 5 | 23406618-24671212 | 0 |
| scf0513114 | 1 | 3341344-4120631 | 3341344-4120631 | 22 | 0 | 26 | 5 | 24681213-25460500 | -2 |
| scf0513187a | 5 | 23614770-25220000 | 23614770-25220000 | 10 | 0 | 46 | 5 | 25470501-27075731 | -1 |
| scf0513087b | 5 | 27431001-27940252 | 27431001-27940252 | 8 | 1 | 8 | 5 | 27085732-27594983 | 0 |
| scf0513078 | 5 | 26864804-27359860 | 26864804-27359860 | 15 | 5 | 21 | 5 | 27604984-28100040 | -1 |
| scf0513106a | 5 | 14077597-14384000 | 14077597-14383714 | 3 | 7 | 0 | 5 | 28110041-28416158 | 0 |
| scf0513080b | 7 | 8182001-8203000 | 8182001-8203000 | 0 | 2 | 0 | 5 | 28426159-28447158 | -2 |
| scf0512965 | 5 | 26222223-26854803 | 26222223-26854803 | 9 | 13 | 8 | 5 | 28457159-29089739 | 1 |
| scf0512952b | 6 | 19620001-19734822 | 19620001-19734822 | 0 | 0 | 4 | 5 | 29099740-29214561 | 0 |
| scf0513168_6b | 6 | 1260501-1266000 | 1260501-1266000 | 0 | 0 | 2 | 5 | 29224562-29230061 | 0 |
| scf0513049 | 6 | 1-203554 | 1-203554 | 0 | 4 | 5 | 6 | 1-203554 | 0 |
| scf0513168_6c | 6 | 1266001-1544462 | 1266001-1544462 | 2 | 14 | 6 | 6 | 213555-492016 | -1 |
| scf0513168_6a | 6 | 213555-1260500 | 213555-1260500 | 50 | 45 | 37 | 6 | 502017-1548962 | -1 |
| scf0513168_1d | 1 | 19539001-19938125 | 19539001-19938125 | 13 | 16 | 21 | 6 | 1558963-1958087 | -1 |
| scf0513168_1b | 1 | 19036001-19533000 | 19036240-19533000 | 3 | 12 | 13 | 6 | 1968088-2464848 | -1 |
| scf0513061 | 6 | 1983910-3376700 | 1983910-3376700 | 10 | 41 | 40 | 6 | 2474849-3867639 | -1 |
| scf0513095a | 1 | 13186592-13768000 | 13186592-13767539 | 8 | 28 | 26 | 6 | 3877640-4458587 | 1 |
| scf0512945 | 6 | 3386701-3723954 | 3386701-3723954 | 5 | 12 | 13 | 6 | 4468588-4805841 | 0 |
| scf0513003 | 6 | 3733955-4095395 | 3733955-4095395 | 0 | 12 | 29 | 6 | 4815842-5177282 | 0 |
| scf0513000 | 6 | 4105396-4200474 | 4105396-4200474 | 0 | 0 | 0 | 6 | 5187283-5282361 | 0 |
| scf0513177 | 6 | 4210475-8315614 | 4210475-8315614 | 74 | 146 | 124 | 6 | 5292362-9397501 | -1 |
| scf0513102 | 6 | 11873481-13152356 | 11873481-13152356 | 13 | 12 | 21 | 6 | 9407502-10686377 | 0 |
| scf0513149 | 6 | 8325615-9795606 | 8325615-9795606 | 10 | 40 | 40 | 6 | 10696378-12166369 | 0 |
| scf0512939 | 6 | 9805607-10064309 | 9805607-10064309 | 3 | 2 | 7 | 6 | 12176370-12435072 | 0 |
| scf0513135b | 5 | 8844001-8865000 | 8844492-8864336 | 1 | 0 | 0 | 6 | 12445073-12464917 | 0 |
| scf0513073 | 6 | 10074310-10978235 | 10074310-10978235 | 6 | 17 | 7 | 6 | 12474918-13378843 | 0 |
| scf0512930 | 6 | 10988236-11056532 | 10988236-11056532 | 0 | 0 | 0 | 6 | 13388844-13457140 | 0 |
| scf0513185a | 6 | 11066533-11199000 | 11066533-11198427 | 2 | 0 | 0 | 6 | 13467141-13599035 | 0 |
| scf0513196b | 6 | 13850001-15497570 | 13850001-15497570 | 27 | 20 | 55 | 6 | 13609036-15256605 | 0 |
| scf0512969 | 6 | 15507571-16107077 | 15507571-16107077 | 18 | 11 | 10 | 6 | 15266606-15866112 | 0 |
| scf0513176b | 6 | 16640001-18575326 | 16645603-18575326 | 34 | 14 | 43 | 6 | 15876113-17805836 | 0 |
| scf0513063 | 6 | 18585327-19091630 | 18585327-19091630 | 20 | 21 | 10 | 6 | 17815837-18322140 | 0 |
| scf0513117 | 6 | 19101631-19408569 | 19101631-19408569 | 4 | 1 | 1 | 6 | 18332141-18639079 | 0 |
| scf0512949 | 6 | 19418570-19476999 | 19418570-19476999 | 4 | 1 | 3 | 6 | 18649080-18707509 | 0 |
| scf0513113b | 7 | 16323001-16658559 | 16323001-16658559 | 9 | 3 | 2 | 6 | 18717510-19053068 | 0 |
| scf0512952a | 6 | 19487000-19620000 | 19487000-19620000 | 0 | 1 | 1 | 6 | 19063069-19196069 | 0 |
| scf0512936 | 6 | 19744823-19953241 | 19744823-19953241 | 3 | 2 | 3 | 6 | 19206070-19414488 | 0 |
| scf0512976 | 6 | 19963242-20015135 | 19963242-20015135 | 0 | 0 | 0 | 6 | 19424489-19476382 | 0 |
| scf0513165 | 6 | 20025136-23577765 | 20025136-23577765 | 17 | 80 | 110 | 6 | 19486383-23039012 | 1 |
| scf0513161 | 6 | 23587766-24765775 | 23587766-24765775 | 29 | 8 | 30 | 6 | 23049013-24227022 | 0 |
| scf0513054 | 6 | 24775776-24952777 | 24775776-24952777 | 3 | 5 | 1 | 6 | 24237023-24414024 | 0 |
| scf0513110 | 6 | 24962778-25980762 | 24962778-25980762 | 15 | 17 | 21 | 6 | 24424025-25442009 | -1 |
| scf0513004b | 4 | 23058001-23936647 | 23058744-23936647 | 14 | 25 | 19 | 6 | 25452010-26329913 | -2 |
| scf0513160_6 | 6 | 26203579-29072900 | 26203579-29072900 | 27 | 49 | 90 | 6 | 26339914-29209235 | 1 |
| scf0513072 | 3 | 3372025-3727613 | 3372025-3727613 | 0 | 13 | 12 | 6 | 29219236-29574824 | 0 |
| scf0512934 | 6 | 29082901-29221817 | 29082901-29221817 | 0 | 3 | 2 | 6 | 29584825-29723741 | 0 |
| scf0513031 | 6 | 29231818-29480786 | 29231818-29480786 | 0 | 1 | 1 | 6 | 29733742-29982710 | 0 |
| scf0513088 | 6 | 29490787-30389603 | 29490787-30389603 | 3 | 20 | 18 | 6 | 29992711-30891527 | 0 |
| scf0513065 | 4 | 1-206650 | 1-206650 | 0 | 3 | 5 | 6 | 30901528-31108177 | 0 |
| scf0512999 | 6 | 30399604-31053569 | 30399604-31053569 | 0 | 14 | 14 | 6 | 31118178-31772143 | 0 |
| scf0512932 | 6 | 31063570-31133148 | 31063570-31133148 | 0 | 5 | 1 | 6 | 31782144-31851722 | 0 |
| scf0512980 | 6 | 31143149-31563805 | 31143149-31563805 | 0 | 23 | 13 | 6 | 31861723-32282379 | 0 |
| scf0512991a | 6 | 34044179-34690000 | 34044179-34690000 | 6 | 23 | 27 | 6 | 32292380-32938201 | -1 |
| scf0512961 | 6 | 36046832-36389855 | 36046832-36389855 | 4 | 3 | 8 | 6 | 32948202-33291225 | 0 |
| scf0513152 | 6 | 31573806-34034178 | 31573806-34034178 | 49 | 78 | 91 | 6 | 33301226-35761598 | 1 |
| scf0512983 | 6 | 1554463-1973909 | 1554463-1973909 | 4 | 0 | 15 | 6 | 35771599-36191045 | 1 |
| scf0513044a | 7 | 16668560-16750000 | 16668560-16750000 | 0 | 1 | 0 | 6 | 36201046-36282486 | 0 |
| scf0513124_4b | 4 | 10011001-10260387 | 10011001-10260386 | 0 | 1 | 9 | 6 | 36292487-36541872 | 1 |
| scf0513124_6 | 6 | 36399856-37673815 | 36400463-37673815 | 13 | 54 | 4 | 6 | 36551873-37825225 | 1 |
| scf0513112a | 6 | 38173164-38722000 | 38173164-38722000 | 13 | 16 | 14 | 6 | 37835226-38384062 | -1 |
| scf0512982 | 6 | 37683816-38163163 | 37683816-38163163 | 5 | 22 | 25 | 6 | 38394063-38873410 | 1 |
| scf0513158_4b | 4 | 25528001-25582000 | 25528794-25582000 | 0 | 1 | 0 | 7 | 1-53207 | 0 |
| scf0513127 | 7 | 5165875-6182419 | 5165875-6182419 | 0 | 15 | 11 | 7 | 63208-1079752 | 0 |
| scf0513185b | 6 | 11199001-11863480 | 11199583-11863480 | 0 | 7 | 4 | 7 | 1089753-1753650 | -2 |
| scf0513179 | 7 | 6192420-7689731 | 6192420-7689731 | 0 | 21 | 19 | 7 | 1763651-3260962 | 0 |
| scf0513080a | 7 | 7699732-8182000 | 7699732-8182000 | 0 | 20 | 13 | 7 | 3270963-3753231 | 0 |
| scf0513080c | 7 | 8203001-8246547 | 8203001-8246547 | 0 | 1 | 1 | 7 | 3763232-3806778 | 0 |
| scf0513069 | 7 | 8256548-8743196 | 8256548-8743196 | 0 | 15 | 8 | 7 | 3816779-4303427 | 0 |
| scf0512946 | 7 | 12414148-13183493 | 12414148-13183493 | 0 | 13 | 14 | 7 | 4313428-5082773 | -1 |
| scf0512990 | 7 | 11408502-12404147 | 11408502-12404147 | 0 | 10 | 18 | 7 | 5092774-6088419 | -1 |
| scf0513136 | 7 | 8753197-9794017 | 8753197-9794017 | 0 | 11 | 55 | 7 | 6098420-7139240 | 0 |
| scf0513156 | 3 | 9809969-11035985 | 9809969-11035985 | 0 | 54 | 31 | 7 | 7149241-8375257 | -2 |
| scf0513153 | 7 | 9804018-11398501 | 9804018-11398501 | 0 | 43 | 39 | 7 | 8385258-9979741 | 0 |
| scf0513089 | 3 | 9045060-9383133 | 9045060-9383133 | 0 | 0 | 1 | 7 | 9989742-10327815 | 0 |
| scf0513170c | 7 | 827001-5155874 | 827026-5155874 | 0 | 136 | 98 | 7 | 10337816-14666664 | -1 |
| scf0513050 | 7 | 13193494-13449415 | 13193494-13449415 | 0 | 0 | 0 | 7 | 14676665-14932586 | 0 |
| scf0513008 | 7 | 13459416-15232378 | 13459416-15232378 | 13 | 62 | 62 | 7 | 14942587-16715549 | 1 |
| scf0513113a | 7 | 15242379-16323000 | 15242379-16323000 | 2 | 21 | 11 | 7 | 16725550-17806171 | -1 |
| scf0513044b | 7 | 16750001-19074300 | 16750001-19074300 | 30 | 76 | 74 | 7 | 17816172-20140471 | 1 |
| scf0513044d | 7 | 19080001-19276478 | 19080001-19276478 | 3 | 5 | 5 | 7 | 20150472-20346949 | 1 |
| scf0513190 | 7 | 19286479-22556666 | 19286479-22556666 | 24 | 89 | 126 | 7 | 20356950-23627137 | -1 |

^a^Scaffold name. Portions of scaffolds that were broken apart are given suffixes of “a,” “b,” etc.

^b^Pseudochromosome in FvH4 reference genome

^c^Range of site positions in FvH4 reference genome

^d^Range of site positions in FvH4 reference genome after trimming Ns on ends

^e^Count of markers in Fvb-s linkage map for this scaffold

^f^Count of markers in Fvb-m linkage map for this scaffold

^g^Count of markers in Fvb-p linkage map for this scaffold

^h^Pseudochromosome in Fvb reference genome

^i^Range of site positions in Fvb reference genome

^j^Orientation of scaffold in Fvb linkage maps (and thus Fvb assembly) relative to FvH4. 1 = same orientation. -1 = reversed orientation. 0 = no information about scaffold orientation in diploid linkage maps, retained FvH4 orientation. -2 = reversed orientation inferred from *Prunus* assembly, diploid linkage maps provide no information on orientation but suggest placement of scaffold on different pseudochromosome, so FvH4 orientation not useful either
