# Supplementary material for: Evolutionary Origins and Dynamics of Octoploid Strawberry Subgenomes Revealed by Dense Targeted Capture Linkage Maps
Source: Genome Biol Evol. 2014 Dec 4;6(12):3295–313. doi: 10.1093/gbe/evu261 (PMC4986458; doi:10.1093/gbe/evu261)
Supplement: Supplementary Data [file supp_evu261_evu261_Table_S1.doc]

**Table S1.** Samples examined in this study.

| **Species^a^** | **Ploidy^b^** | **Abbreviation^c^** | **Accession(s)^d^** | **NCBI^e^** | **Depth^f^** | **Mb^g^** | **Offspring (Analyzed)^h^** | **LG SNPs^i^** | **Reference** |
| --- | --- | --- | --- | --- | --- | --- | --- | --- | --- |
| *Fragaria vesca* ssp. *vesca* | 2X | FvH4 | PI 551572 | GCF_000184155.1 | na | na | na | na | (36) |
| *Fragaria vesca* ssp. *bracteata* | 2X | Fvb-m, Fvb-p | PI 664465 (MRD30), MRD60 | SRP022950 | na | na | 48 (48) | 6575 | (39) |
| *Fragaria vesca* ssp. *bracteata* | 2X | Fvb-s | LNF23 | SRP050248 | na | na | 43 (41) | 1825 | this publication |
| *Fragaria chiloensis* | 8X | Fchil | GP33, SAL3 | SRP050248 | 15.3x | 167.5 | 46 (42) | 2542 | this publication, (46) |
| *Fragaria virginiana* ssp. *virginiana* | 8X | Fvirg | PI 657872, PI 657873 | SRP050248 | 29.1x | 171.9 | 73 (67) | 3875 | this publication, (43) |
| *Fragaria x ananassa* | 8X | Fanan | 02–19, Sachinoka, Kaorino, Akihime, 0212921 | na | na | na | na | 777 | (34) |
| *Fragaria bucharica* | 2X | F. bucharica | PI 551853 (CFRA 522) | GCA_000511995 | na | na | na | na | (35) |
| *Fragaria iinumae* | 2X | F. iinumae | KAR-JP-251 | SRX733177 | 3.5x | 121.4 | na | na | this publication |
| *Fragaria mandshurica* | 2X | F. mandshurica | PI 657855 (CFRA 1947) | SRX733178 | 6.1x | 142.1 | na | na | this publication |
| *Fragaria nipponica* | 2X | F. nipponica | nipponica1 | GCA_000512025 | na | na | na | na | (35) |
| *Fragaria viridis* | 2X | F .viridis | PI 616609 (CFRA 1256) | SRX733175, SRX733176 | 2.4x | 63.1 | na | na | this publication |
| *Rubus coreanus* | 2X | R. coreanus | na | SRX347804 | na | na | na | na | (65) |

^a^Species name

^b^Ploidy level. 2X = diploid; 8X = octoploid

^c^Abbreviation as it appears in Figure 5 and elsewhere

^d^Accessions of plant tissue. PI = USDA Plant Introduction numbers; CFRA = USDA Corvallis Germplasm Repository *Fragaria* numbers.

^e^SRA or Assembly accession numbers

^f^For complete genomes sequenced in this study, mean coverage over 208.9 Mb of Fvb

^g^For complete genomes sequenced in this study, total number of Fvb sites, in Mb, with nonzero coverage

^h^For linkage crosses, total number of offspring sequenced, and (in parentheses), total number of offspring analyzed after excluding those with low or poor-quality sequence

^i^Markers used in linkage mapping. SNPs for octoploids in this study, SNPs or small indels for *Fragaria vesca* ssp. *bracteata*, SSRs for *Fragaria* x *ananassa*
